# The effects of plyometric training on physical fitness in adolescent team sports: a systematic review and meta-analysis

**DOI:** 10.3389/fphys.2026.1760239

**Published:** 2026-01-21

**Authors:** Fengming Zhang, Yang Liu, Jiale Liu, Oleksandr Yeremenko, Lei Shi

**Affiliations:** 1 National University of Physical Education and Sport of Ukraine, Kyiv, Ukraine; 2 Shandong Sport University, Jinan, China

**Keywords:** adolescents, athletic performance, physical fitness, plyometric training, team sports

## Abstract

**Objectives:**

This systematic review and meta-analysis examined the effects of plyometric training (PT) on the physical fitness of adolescent team-sport athletes.

**Methods:**

We systematically searched the PubMed, Web of Science, Scopus, and Embase databases. The methodological quality of the studies was evaluated using the Cochrane Risk of Bias Tool (ROB-2). Meta-analyses were conducted using RevMan 5.4 and STATA 15.0.

**Results:**

A total of 31 studies involving 1,033 athletes (906 males and 127 females) were ultimately included. PT improved jump performance, including countermovement jump (ES = 0.89), countermovement jump with arms (ES = 1.00), squat jump (ES = 0.48), and standing long jump (ES = 1.10). PT also improved linear sprint over ≤10-m (ES = −0.59), 20-m (ES = −0.42), and 30-m (ES = −0.97), and improved change-of-direction (ES = −0.73).

**Conclusion:**

Plyometric training can significantly improve the jumping performance, linear sprint and change-of-direction in adolescent team-sport athletes. Athletes aged 16–18.99 years may show larger improvements, and interventions lasting ≥8 to <10 weeks may be associated with more consistent gains, particularly for Countermovement Jump, SJ, ≤10-m linear sprint, and 20-m linear sprint. In contrast, increasing the total number of jumps was not consistently associated with greater training effects.

**Systematic Review Registration:**

https://www.crd.york.ac.uk/prospero/, identifier CRD420251034889.

## 1 Introduction

Team sports such as football, basketball, handball, and volleyball are high-intensity intermittent sports (Stølen et al., 2005; Duncan et al., 2006; Ziv and Lidor, 2009; Abdelkrim et al., 2010), requiring athletes to repeatedly perform high-intensity explosive movements such as jumping, sprinting, sudden stops, and changes of direction, and high-intensity physical contact during the game (Ostojic et al., 2006; Faude et al., 2012; Passos et al., 2017; Taylor et al., 2017). Excellent physical fitness, such as strength, speed, and change of direction, is essential for executing explosive movements and for athletes to maintain peak performance and success in high-level competitions (Stølen et al., 2005; Ostojic et al., 2006; Wagner et al., 2014; Mancha-Triguero et al., 2019). During the critical period of neuromuscular development in adolescence, targeted physical training can not only effectively improve physical fitness, such as strength, speed, and agility, but also lay the foundation for an athletic career (Moran J. et al., 2017; Moran J. J. et al., 2017; Radnor et al., 2018). Jumping ability, speed, and change of direction are the basis for assessing athletic potential and future development into high-level athletes during the talent selection process for adolescents (Burgess and Naughton, 2010; Unnithan et al., 2012; Han et al., 2023; Kelly, 2023; Sanpasitt et al., 2023). Therefore, designing effective physical training methods for teenagers is very important.

Traditional resistance training, plyometric training (PT), compound training, and sprint training are commonly used effective training methods for improving physical fitness (MacDonald et al., 2012; Morris et al., 2022). Numerous studies have demonstrated that, compared with traditional resistance training, plyometric training may provide greater improvements in explosive power, sprint speed, and change-of-direction (Rædergård et al., 2020; Luo et al., 2025). PT utilizes the physiological advantages of stretch-shortening cycles (SSC), it employs a muscle contraction pattern characterized by a rapid eccentric pre-stretch followed by a rapid concentric contraction (Lloyd et al., 2011; Davies et al., 2015). This muscle contraction pattern is closer to the explosive movement patterns of jumping and sprinting in team sports such as basketball, football, and handball, thus improving performance in actual sports (Slimani et al., 2016; Wang et al., 2024). This improvement is primarily achieved through long-term training, leading to various adaptive mechanisms such as muscle fiber hypertrophy, enhanced motor unit recruitment, increased tendon stiffness, and improved intramuscular and intermuscular coordination (Komi, 2003; Fouré et al., 2010; Taube et al., 2012; Chu and Myer, 2013).

Numerous meta-analyses of PT have confirmed its effectiveness in improving jumping performance, linear sprinting, and change-of-direction. These studies either included both adults and adolescents or only included general adolescents, rather than trained adolescent athletes (de Villarreal et al., 2012; Oxfeldt et al., 2019; Chen et al., 2023a; Sun et al., 2025). Existing evidence suggests that untrained adolescents, due to their lower baseline fitness levels, show greater improvement than trained adolescents (Behm et al., 2017). Furthermore, adolescents are in a critical stage of growth and development, and their neuromuscular systems, hormonal and metabolic levels, and recovery and adaptation abilities differ from those of adults (Lloyd et al., 2015).Therefore, applying evidence from adults or untrained adolescents to guide PT programming in adolescent team-sport athletes may not yield optimal training adaptations.

Existing systematic reviews and meta-analyses have summarized the effects of PT in the adolescent population, but they mostly focus on specific groups and do not cover all the outcome indicators comprehensively (Chen et al., 2023b; Chen et al., 2024). Currently, there is a lack of a systematic evaluation that targets adolescent team sport athletes and integrates key physical fitness indicators such as jumping, different distances of linear sprints, and change-of-direction. Therefore, this meta-analysis aims to explore the impact of PT on the physical fitness of adolescent team athletes and to conduct moderating-variable analyses, including age, gender, training program, and training volume, to investigate the potential influence of these factors on the effectiveness of training. The aim is to establish an evidence base for the scientific development of safe and efficient PT programs for adolescent team sports.

## 2 Methods

This meta-analysis and systematic review adhered to the Preferred Reporting Items for Systematic Reviews and Meta-Analyses (PRISMA) (Moher et al., 2009). It was registered in PROSPERO under the registration number CRD420251034889.

### 2.1 Information sources and search strategy

A comprehensive search was conducted across the Scopus, Web of Science, PubMed, and Embase databases. The initial search was conducted on 23 April 2025, and was updated on 6 November 2025. Database searches used keywords combined with MeSH terms. Search terms included: “Stretch-Shortening Exercise” OR “Stretch Shortening Cycle” OR “plyometric training” OR plyometric OR plyometrics OR “jump training” OR “jump exercise” OR “ballistic training” OR “drop jump” OR “depth jump” AND “basketball” OR “soccer” OR “football” OR “handball” OR “volleyball” OR “rugby” OR “team sport”. The search was limited to titles and abstracts, with no restrictions applied to publication region, year, or language. We also searched PROSPERO and the Cochrane Database of Systematic Reviews for relevant protocols to determine whether they had been published.

### 2.2 Eligibility criteria

Eligibility criteria were defined according to the PICOS framework and are summarized in Table 1. The age range followed the World Health Organization definition of adolescents (10–19 years) (Organization, 2023).

**TABLE 1:** Eligibility criteria (PICOS).

Category	Inclusion criteria	Exclusion criteria
Population (P)	Healthy adolescent team-sport athletes aged 10–19 years (e.g., basketball, football/soccer, handball, volleyball, *etc.*); this age range is consistent with the World health Organization (WHO) definition of adolescents	Adolescent team athletes who are over the age limit or have health problems such as injuries or recent surgery
Intervention (I)	At least 4 weeks of PT. The experimental group must add PT to usual sport-specific training; PT primarily involves SSC-based lower-limb explosive jump drills (e.g., jumps/hops/bounds, drop/depth jumps, reactive jumps). The control group receives only conventional specialized training, similar to that of the experimental group, without any plyometric training	Duration under 4 weeks; Interventions combining PT with other training modalities (e.g., strength training, sprint training, aerobic training, agility training, or upper-body plyometric training)
Comparison (C)	Active control: Usual team-sport training or general physical conditioning, without additional plyometric training	Lack of active control
Outcome (O)	Include results from at least one physical fitness measure listed below: Countermovement jump (CMJ), countermovement jump with arms (CMJA), standing long jump (SLJ), ≤10-m linear sprint, 20-m linear sprint, 30-m linear sprint, or change-of-direction (COD)	Incomplete data reporting; presented solely in graphical format with no extractable data
Study design (S)	Randomized controlled trial (RCT)	Non-randomized trials; non-controlled studies; single-group pre-post designs; observational studies; and secondary research (reviews, meta-analyses, and study protocols)

### 2.3 Selection process

Duplicate references were identified and removed by one reviewer (FZ) using EndNote 21 (Clarivate Analytics, Philadelphia, Pennsylvania, USA). Two researchers (FZ and YL) then independently screened titles, abstracts, and full texts against the predefined criteria.

### 2.4 Data extraction

Basic information of the literature was extracted independently by one author (FZ), including: (1) author and publication year; (2) age and gender of the subjects; (3) sample size; (4) sport and athlete level; (5) intervention measures; (6) training duration, training frequency, and training volume; and (7) outcome indicators. The results were reviewed by a second author (YL). We first attempted to obtain missing or unclear data by directly emailing the corresponding authors. All discrepancies between reviewers were then resolved through discussion. For any persisting disagreements, a final decision was made by a designated senior reviewer (LS). To avoid overestimating the sample size, if a control group in a study is compared with multiple experimental groups, the sample size of the control group should be divided by the number of comparisons for allocation. Biological maturity information was extracted and summarized descriptively, with emphasis on the reported maturity metrics. Athlete level was reclassified in a standardized manner using the McKay Participant Classification Framework (PCF; Tier 0–5) (McKay et al., 2021). When multiple COD tests were reported in a study, only the longest test time was included in the analysis, defined as the COD protocol with the greatest total test distance or the highest number of directional changes.

### 2.5 Risk of bias assessment and certainty of evidence

Two assessors (FZ and JL) independently assessed risk of bias using the Cochrane RoB 2.0 tool. The assessment covered five domains: (D1) Randomisation process, (D2) deviations from intended interventions, (D3) missing outcome data, (D4) measurement of the outcome, and (D5) selection of the reported result. Judgements were made for each domain and overall, as low risk, some concerns, or high risk. The certainty of evidence for each primary outcome was assessed using the GRADE approach. As all included studies were randomized controlled trials, certainty started at high and was downgraded when applicable across the following domains: risk of bias, inconsistency, indirectness, imprecision, and other considerations (e.g., publication bias). The overall certainty for each outcome was rated as high, moderate, low, or very low (Guyatt et al., 2011). Disagreements were resolved by discussion, with arbitration by a third assessor when necessary.

### 2.6 Statistical analysis

The meta-analyses were conducted using Review Manager V.5.4.0 and Stata 15.0. A total of eight meta-analyses were performed: (1) CMJ, (2) CMJA, (3) SJ, (4) SLJ, (5) ≤10-m linear sprint, (6) 20-m linear sprint, (7) 30-m linear sprint, (8) COD. A meta-analysis was conducted when at least three independent studies reported the same outcome measure (Borenstein et al., 2021). The effect size (ES) is represented by Hedge’s g and calculated by the mean and standard deviation of each dependent variable before and after training. For time-based outcomes, negative effect sizes represent improvements in performance. Given the expected between-study differences in participants, training programmes, and testing protocols, a random-effects model was used to pool effect sizes. Pooled effects are presented as Hedges’g with 95% confidence intervals (Deeks et al., 2019). The effect size is explained by the following criteria: trivial (<0.2), small (0.2-0.6), moderate (>0.6-1.2), large (>1.2-2.0), very large (>2.0-4.0), and extremely large (>4.0) (Hopkins et al., 2009). Heterogeneity among studies was assessed using the I^2^ statistic, categorized as low (<25%), moderate (25%–75%), or high (>75%) (Higgins and Thompson, 2002). Egger’s test was used to assess publication bias. When publication bias was detected, the trim-and-fill method was used (Duval and Tweedie, 2000). Sensitivity analysis was employed to ensure the robustness of the meta-analysis results. p < 0.05 was set as the threshold for statistical significance.

To explore potential sources of heterogeneity, subgroup analyses and meta-regression analyses were conducted. Age, training duration, and total number of jumps were taken as moderating variables. Specifically, age groups followed the WHO age-based developmental stage classification described in: 10–12.99 (pre-PHV), 13–15.99 (mid-PHV), and 16–18.99 years (post-PHV), which reflects chronological age rather than directly assessed maturity. The training duration and the total number of jumps lack standardized classifications. To ensure the subgroup analysis has sufficient statistical power, we grouped them according to the distributions observed in the included studies. Meta-regression analysis was conducted when at least 10 studies reported the same outcomes (Cumpston et al., 2019).

## 3 Results

### 3.1 Study selection

**FIGURE 1:**
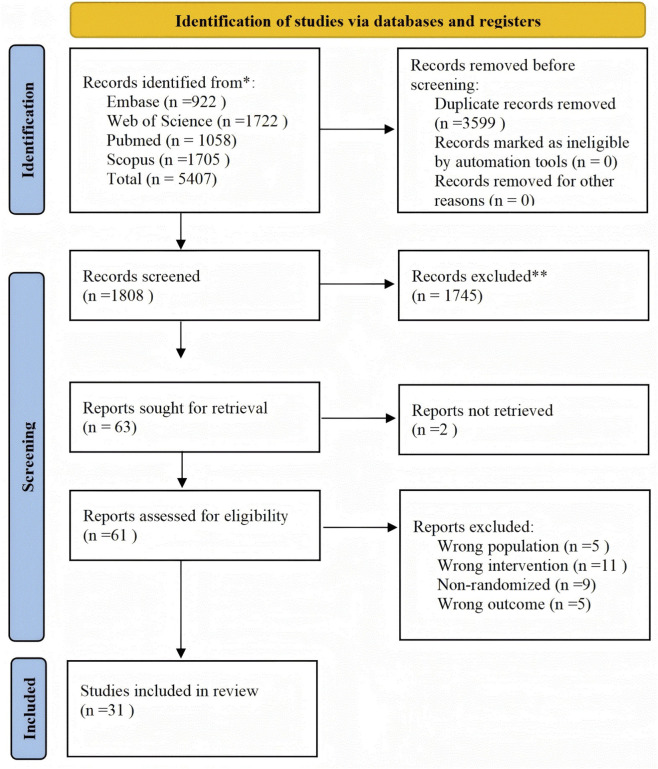
Systematic review search and screening procedure.

A preliminary literature search yielded 5,407 articles. After deleting duplicate literature, an initial screening was performed based on the title and abstract, followed by downloading and reading the full text. Finally, 31 studies met the inclusion criteria (Sankey et al., 2008; Sedano et al., 2011; Ozbar et al., 2014; Zribi et al., 2014; Attene et al., 2015; Hammami et al., 2016; Asadi et al., 2018; Hernández et al., 2018; Ramirez-Campillo et al., 2018; Fathi et al., 2019; Jlid et al., 2019; Meszler and Váczi, 2019; Ramirez-Campillo et al., 2019; Drouzas et al., 2020; Negra et al., 2020; Ramirez-Campillo et al., 2020a; Ramirez-Campillo et al., 2020b; Vera-Assaoka et al., 2020; de Villarreal et al., 2021; Noutsos et al., 2021; Padrón-Cabo et al., 2021; Palma-Muñoz et al., 2021; Paes et al., 2022; Gaamouri et al., 2023; Aztarain-Cardiel et al., 2024; BOGIATZIDIS et al., 2024; Haghighi et al., 2024; Liu et al., 2024; Sammoud et al., 2024; Türkarslan and Deliceoglu, 2024; Öztürk et al., 2025). The complete literature screening process is summarized in Figure 1.

### 3.2 Characteristics of participants and interventions

Characteristics of study participants and intervention protocols are detailed in Table 2.

**TABLE 2:** Data extraction from selected article.

References	Participants characteristics	Intervention	Control	Characteristics of intervention				Measurements	Outcome	
				Train content	L/F/D	Volume	Season		Time	Groups
Aztarain-Cardiel et al. (2024)	N = 31; TB:7.6 ± 1.3years; S:M E.G.,1: A = 14.5 ± 1.9years, H = 181.5 ± 6.9 cm BM = 69 ± 11.4 kg E.G.,2: A = 15.1 ± 2.2years, H = 181.3 ± 10.3 cm BM = 70.2 ± 13.2kg; CG: A = 15.3 ± 0.2years, H = 187.1 ± 8.1 cm BM = 74.8 ± 13.4 kg PL: Tier 3 (highly trained/National) Maturity metric:NR	PT	Basketball	MIX	L: 6 weeks F: 2 sessions/week D:NR	E.G.,1:80-128 per session 1,184 in total, E.G.,2:40-64 per session 592 in total	In-season	CMJ, SJ, SLJ, 20 m Sprint, COD ability (V-Cut)	E.G.,1 and, E.G.,2: CMJ, SJ, SLJ ↑, others ↔; CG: all ↔	CMJ, SJ ↑, others ↔ in, E.G.,1 and, E.G.,2 vs. CG
Zribi et al. (2014)	N = 51; TB:2.5 ± 0.5; S:M E.G.,: A = 12.1 ± 0.6years, H = 155.5 ± 6.7 cm BM = 41.1 ± 8.2 kg CG: A = 12.2 ± 0.4years, H = 154.8 ± 7.6 cm , BM = 41.2 ± 7.8 kg PL: Tier 2 (Trained/Developmental) Maturity metric:Tanner	PT	Basketball	V-JUMP	L: 9 weeks F: 2 sessions/week D:15–25 min	60–100 per session 1,440 in total	In-season	5 m,30 m sprint, CMJ, SJ, CMJA, SLJ (5jumps)	E.G.,:5 m,30 m Sprint, CMJ, SJ, CMJA ↑, others ↔; CG: All ↔	5 m,30 m Sprint, CMJ, SJ, CMJA↑, others ↔ in, E.G., vs. CG
Palma-Muñoz et al. (2021)	N = 22; TB:NR; S:M A = 13.5 ± 2.0years, H = 160.1 ± 10.9 cm BM = 62.1 ± 13.5 kg PL: Developmental PL: Tier 2 (Trained/Developmental) Maturity metric:NR	PT	Basketball	MIX	L: 6 weeks F: 2 sessions/week D:15–26 min	E.G.,1:60 per session 720 in total, E.G.,2:60–84 per session 864 in total	In-season	CMJ, CMJA, SLJ, 10 m Sprint, COD ability (T-test)	E.G.,1: COD ↑, others ↔; E.G.,2: CMJ, CMJA, SLJ, 10 m Sprint, COD ↑, others ↔ CG: All ↔	All ↔ in, E.G.,1 vs. CG; SLJ, CMJ, CMJA ↑, others ↔ in, E.G.,2 vs. CG
Attene et al. (2015)	N = 36; TB:NR; S:FM A = 14.9 ± 0.9years, H = 160.4 ± 7.6 cm BM = 54.0 ± 8.7 kg PL: Tier 3 (highly trained/National) Maturity metric:NR	PT	Basketball	MIX	L: 6 weeks F: 2 sessions/week D:20 min	67-126 per session 1,120 in total	NR	CMJ, SJ	E.G.,: All ↑ CG: All ↔	CMJ and SJ ↑ in, E.G., vs. CG
de Villarreal et al. (2021)	N = 20; S:M, A = 14.2 ± 1.3years H = 1.68 ± 0.17cm, BM = 52.5 ± 4.2 kg E.G.,: A = 13.57 ± 1.39years, TB = 4.85 ± 1.86 years CG: A = 14.66 ± 0.86years, TB = 3.44 ± 1.50 years PL: Tier 2 (Trained/Developmental) Maturity metric:Maturity offset/PHV	PT	Basketball	MIX	L: 7 weeks F: 2 sessions/week D:20 min	140-180 per session 1,340 in total	In-season	CMJ, CMA, COD ability (Zig-zag 10 m),10 m,20 m Sprint	E.G.,: CMJ, CMJ↑, others ↔; CG: all ↔	COD↑, Others↔ in, E.G., vs. CG
Paes et al. (2022)	N = 34; TB:NR; S:M and FM E.G.,1: A = 15.83 ± 0.75years, H = 183 ± 7 cm BM = 70.78 ± 11.83kg; E.G.,2: A = 14.45 ± 0.69years, H = 160 ± 7 cm BM = 53.72 ± 9.01 kg CG1: A = 15.43 ± 1.13years, H = 174 ± 13 cm BM = 72.94 ± 24.13 kg CG2: A = 15.30 ± 1.16years, H = 163 ± 8 cm BM = 59.98 ± 16.74 kg PL: Tier 3 (highly trained/National) Maturity metric:NR	PT	Basketball	MIX	L: 6 weeks F: 2 sessions/week D:30–60 min	50-100 per session 860 in total	Pre-season	20 m Sprint, COD ability (Illinois agility test)	E.G.,: All ↑ CG: All ↔	NR
Hernández et al. (2018)	N = 19; TB:5 years at least; S:M E.G.,1: A = 10.0 ± 1.5 years = 141 ± 9 cm E.G.,2: A = 11.0 ± 1.7years, H = 142 ± 1 cm CG: A = 9.7 ± 2.0years, H = 144 ± 8 cm PL: Tier 2 (Trained/Developmental) Maturity metric:NR	PT	Basketball	MIX	L: 7 weeks F: 2 sessions/week D:NR	71-170 per session 1,556 in total	In-season	CMJ,20 m,30 m sprint, COD (T-test)	E.G.,1 and, E.G.,2: All ↑ CG: All ↔	All ↑ in, E.G.,1 and, E.G.,2 VS CG
Haghighi et al. (2024)	N = 16; S:FM E.G.,: A = 14.6 ± 1.5years, H = 168.3 ± 8.7 cm BM = 61.7 ± 10.3kg, TB = 5.1 ± 1.1year CG: A = 15.1 ± 1.8years, H = 165.8 ± 9.7 cm BM = 56.7 ± 13.6kg, TB = 5.1 ± 1.2years PL: Tier 3 (highly trained/National) Maturity metric:Tanner	PT	Basketball	MIX	L: 6 weeks F: 2 sessions/week D:NR	63-108 per session 1,016 in total	Pre-season	20 m sprint, COD (lane agility drill)	E.G.,: All ↑ CG: All ↔	20mSprint↑, Others↔ in, E.G., vs. CG
Meszler and Váczi (2019)	N = 18; TB:5 years at least; S:FM E.G.,: A = 15.8 ± 1.2years, H = 176.4 ± 8.6 cm BM = 63.5 ± 8.6 kg CG: A = 15.7 ± 1.3years, H = 177.5 ± 7.4 cm , BM = 66.1 ± 8.9 kg PL: Tier 2 (Trained/Developmental) Maturity metric:NR	PT	Basketball	MIX	L: 7 weeks F: 2sessions /Week D: 20 min	40–100 per session 1,027 in total	In-season	CMJ, COD (T-test, IAT)	E.G.,: CMJ↓, others ↔; CG: All ↔	NR
Asadi et al. (2018)	N = 60; TB:2 years at least; S:M E.G.,1: A = 11.5 ± 0.8years, H = 138.3 ± 6.0 cm BM = 31.0 ± 3.9 kg E.G.,2: A = 14.0 ± 0.7years, H = 154.5 ± 6.5 cm BM = 43.5 ± 6.3 kg E.G.,3: A = 16.6 ± 0.6years, H = 171.5 ± 6 cm BM = 60.6 ± 6.7 kg CG1: A = 11.7 ± 0.4years, H = 137.4 ± 5.0 cm BM = 33.1 ± 3.2 kg CG2: A = 14.2 ± 0.6years, H = 150.1 ± 7.2 cm BM = 41.2 ± 7.6 kg CG3: A = 16.6 ± 0.6years, H = 176.4 ± 5.0 cm BM = 62.4 ± 7.2 kg PL: Tier 3 (highly trained/National) Maturity metric:Maturity offset/PHV	PT	Soccer	V-JUMP	L: 6 weeks F: 2 sessions /Week D: 30–40 min	60 per session 720 in total	NR	CMJ, SLJ,20 m sprint	E.G.,: all↑ CG: All ↔	NR
Hammami et al. (2016)	N = 28; TB:NR; S:M E.G.,: A = 15.7 ± 0.2years, H = 176 ± 6 cm BM = 59.0 ± 6.5kg; CG: A = 15.8 ± 0.2years, H = 176 ± 6 cm BM = 58.2 ± 5.0 kg PL: Tier 2 (Trained/Developmental) Maturity metric:Tanner	PT	Soccer	V-JUMP	L: 8 weeks F: 2 sessions /Week D: 20 min	35-70 per session 722 in total	Pre-season	5 m,10 m,20 m,30 m sprint, COD (the sprint 9-3-6-3-9 m withS180°turns and backward and forward running)	E.G.,:5 m,10 m,20 m sprint ↑ Others ↔; CG: All ↔	5 m Sprint↑, others ↔ in, E.G., VS CG
Negra et al. (2020)	N = 24; TB: 5.0 ± 1.3; S:M, E.G.,: A = 12.7 ± 0.2years, H = 158.6 ± 4.5 cm BM = 43.7 ± 5.7 kg CG: A = 12.70 ± 0.2years, H = 152.0 ± 6 cm BM = 39.9 ± 5.8 kg PL: Tier 2 (Trained/Developmental) Maturity metric:Maturity offsetd/APHV	PT	Soccer	MIX	L: 8 weeks F: 2 sessions/week D: 35 min	50-120 per session 723 in total	In-season	20 m sprint, COD (T-test)	E.G.,: All ↑ CG: COD, others ↔	20 m Sprint, COD ↑ in, E.G., VS CG
Ozbar et al. (2014)	N = 18; S:FM E.G.,: A = 18.3 ± 2.6years, H = 163.1 ± 5.3 cm BM = 58.8 ± 7.8kg, TB = 4.2 ± 0.9years CG: A = 18.0 ± 2.0years, H = 159.4 ± 5.1 cm BM = 56.7 ± 13.6kg, TB = 4.3 ± 0.8 years PL: Tier 2 (Trained/Developmental) Maturity metric:Maturity offsetd/APHV	PT	Soccer	MIX	L: 8 weeks F: 1 session /Week D:30–40 min	90-220 per session 1,210 in total	In-season	CMJ, SLJ,20 m Sprint	E.G.,: All ↑ CG: 20-M sprint ↓, others ↑	All ↑ in, E.G., vs. CG
Padrón-Cabo et al. (2021)	N = 20; S:M, TB: 5.52 ± 1.21year E.G.,: A = 12.60 ± 0.70years, H = 161.20 ± 10.91cm, BM = 48.9 ± 6.44 kg CG: A = 12.39 ± 0.56years, H = 158.0 ± 8.50 cm BM = 46.75 ± 7.40 kg PL: Tier 2 (Trained/Developmental) Maturity metric:NR	PT	Soccer	H-JUMP	L: 6 weeks F: 2 sessions /Week D:20–35 min	24-56 per session 512 in total	In-season	CMJ, CMJA, SJ, COD (IAT)	E.G.,:CMJ, CMJA ↑, others ↔; CG: All ↔	CMJA, others ↔ in, E.G., vs. CG
Sedano et al. (2011)	N = 22; S:M E.G.,: A = 18.4 ± 1.1year, H = 174 ± 3.1 cm BM = 70.7 ± 0.8 kg, TB = 4.2 ± 0.6 years CG: A = 18.2 ± 0.9years, H = 175 ± 4.5 cm BM = 71.1 ± 1.2kg, TB = 4.1 ± 1.0 years PL: Tier 3 (highly trained/National) Maturity metric:NR	PT	Soccer	MIX	L: 10 weeks F: 3 sessions /Week D:20–36 min	80-130 per session 2,880 in total	In-season	CMJ, CMJA, SJ,10 m sprint	E.G.,: All ↑ CG: All ↔	NR
Ramirez-Campillo et al. (2020a)	N = 38; S:M E.G.,1: A = 16.9 ± 0.7years, H = 172.3 ± 4.9 cm BM = 64.9 ± 4.8kg, TB = 6.8 ± 1.5years E.G.,2: A = 17.1 ± 0.3years, H = 174.9 ± 4.5 cm BM = 65.4 ± 3.4kg, TB = 7.7 ± 2.3years CG: A = 17.1 ± 0.5years, H = 174.9 ± 4.4 cm BM = 66.8 ± 3.1 kg, TB = 7.3 ± 1.8years PL: Tier 3 (highly trained/National) Maturity metric:Tanner	PT	Soccer	MIX	L: 7 weeks F: 2 sessions /Week D: 20 min	E.G.,1and, E.G.,2: 69-240 per session 2,334 in total	In-season	CMJ, SLJ, SJ,20 m sprint, COD (Illinois)	E.G.,: All ↑ CG: All ↑	All ↑ in, E.G.,1 vs. CG; CMJ, SLJ ↑, others ↔ in, E.G.,2 vs. CG
Jlid et al. (2019)	N = 28; S:M E.G.,: A = 11.8 ± 0.4years, H = 143 ± 10 cm BM = 34.2 ± 3.6 kg, TB = 3.8 ± 0.4 years CG: A = 11.6 ± 0.5years, H = 142 ± 4 cm BM = 36.5 ± 5.1 kg, TB = 3.6 ± 0.5 years PL: Tier 2 (Trained/Developmental) Maturity metric:NR	PT	Soccer	MIX	L: 6 weeks F: 2 sessions /Week D: 20–25 min	54-124 per session 1,596 in total	Pre-season	CMJ, SJ, COD (T-test)	E.G.,: All ↑ CG: All ↔	All ↑ in, E.G., vs. CG
Drouzas et al. (2020)	N = 68; S:M E.G.,1: A = 9.9 ± 1.8years, H = 142.2 ± 8.7 cm BM = 39.3 ± 8.2kg, TB = 4.3 ± 2.0years E.G.,2: A = 10.0 ± 0.5years, H = 139.2 ± 7.0 cm BM = 36.1 ± 7.8kg, TB = 3.5 ± 1.5years CG: A = 10.2 ± 1.7years, H = 141.6 ± 10.7 cm BM = 38.5 ± 3.1kgTB = 3.7 ± 1.2years PL: Tier 3 (highly trained/National) Maturity metric:NR	PT	Soccer	MIX	L: 10 weeks F: 2 sessions /Week D: 15 min	E.G.,1 and, E.G.,2: 60-120 per session 1,440 in total	Pre-season	CMJ, SJ, SLJ,5 m,10 m,20 m sprint, COD (T-test)	E.G.,1: SLJ, CMJ,5 m Sprint, COD ↑, others ↔ E.G.,2: SLJ, COD ↑, others ↔ C: SLJ, COD ↑, others ↔	NR
Bogiatzidis et al. (2024)	N = 30; S:M E.G.,1: A = 14.0 ± 0.8years, H = 172 ± 6 cm BM = 63.76 ± 8.50kg, TB = 6.2 ± 1.8years E.G.,2: A = 14.3 ± 0.8years, H = 175 ± 8 cm BM = 66.48 ± 12.68kg, TB = 7.5 ± 3.2years CG: A = 14.2 ± 0.7years, H = 170 ± 8 cm BM = 59.96 ± 11.05kg, TB = 5.1 ± 2.6years PL: Tier 3 (highly trained/National) Maturity metric:NR	PT	Soccer	E.G.,1: V-JUMP E.G.,2: H-JUMP	L:12 weeks F: 2 sessions /Week D: NR	40-65 per session 1,410 in total	In-season	CMJ, SJ	E.G.,1:E.G.,: All ↑ E.G.,2:SJ ↑, others ↔ CG: All ↔	All ↑ in, E.G.,1 and, E.G.,2 vs. CG
Liu et al. (2024)	N = 51; TB = 4.9 ± 2.9 years; S:M A = 16.3 ± 0.6years, H = 173.5 ± 2.9 cm BM = 62.3 ± 2.1 kg PL: Tier 2 (Trained/Developmental) Maturity metric:NR	PT	Soccer	H-JUMP	L: 8 weeks F: 2 sessions /Week D: E.G.,1: 14 min E.G.,2: 11 min	E.G.,1: 34-48 per session 656 in total E.G.,2: 9-14 per session 328 in total	NR	CMJ, SJ, 10 m sprint	E.G.,: All ↑ CG: All ↔	All ↑ in, E.G.,1 and, E.G.,2 vs. CG
Sammoud et al. (2024)	N = 27; TB = 5.0 ± 1.1 years S:M E.G.,: A = 12.7 ± 0.2 years, H = 155.8 ± 7.4 cm, BM = 47.9 ± 7.3 kg CG: A = 11.8 ± 0.4 years, H = 148.1 ± 7.3 cm, BM = 39.4 ± 5.3 kg PL: Tier 2 (Trained/Developmental) Maturity metric:Maturity offsetd/APHV	PT	Soccer	MIX	L: 6 weeks F: 2 sessions/week D: 35–40 min	50-120 per session 1,304 in total	In-season	CMJ, SLJ, COD (505 test)	E.G.,: All ↑ CG: All ↔	All ↑ in, E.G., vs. CG
Türkarslan and Deliceoglu (2024)	N = 26; TB:NR, S:M E.G.,: A = 15.00 ± 0.22 years, H = 173.23 ± 6.45 cm, BM = 62.92 ± 6.51 kg CG: A = 15.08 ± 0.23 years, H = 172.54 ± 5.21 cm, BM = 62.38 ± 4.59 kg PL: Tier 2 (Trained/Developmental) Maturity metric:NR	PT	Soccer	MIX	L: 8 weeks F: 2 sessions /Week D: 20–26 min	96-180 per session 1,680 in total	NR	CMJ, SJ, 30 m sprint, COD (T-test)	E.G.,: All ↑ CG: COD, 30 m Sprint ↑, others ↔	All ↑ in, E.G., vs. CG
Öztürk et al. (2025)	N = 24; S:M E.G.,1: A = 18.12 ± 0.35years, H = 174 ± 2cm, BM = 73.5 ± 3.62 kg TB = 8.12 ± 0.64years E.G.,2: A = 18.50 ± 0.53years, H = 175 ± 4 cm BM = 72.25 ± 3.32 kg, TB = 8.00 ± 0.92 years CG: A = 18.12 ± 0.35years, H = 177 ± 7cm, BM = 73.50 ± 5.58 kg, TB = 7.75 ± 0.70 years PL: Tier 3 (highly trained/National) Maturity metric:NR	PT	Soccer	MIX	L: 8 weeks F: 2 sessions /Week D: NR	NR per session 1,000 in total	In-season	CMJ, 10m, 20m, 30 m sprint, COD (zigzag)	E.G.,1 and, E.G.,2: All ↑ CG: All ↔	All ↑ in, E.G.,1 and, E.G.,2 vs. CG
Ramirez-Campillo et al. (2019)	N = 39; TB: 2 years at least, S:M E.G.,: A = 13.2 ± 1.8years, H = 154 ± 11 cm BM = 48.6 ± 9.9 kg CG: A = 13.5 ± 1.9years, H = 155 ± 11 cm BM = 49.1 ± 12.0 kg PL: Tier 2 (Trained/Developmental) Maturity metric:Tanner	PT	Soccer	MIX	L: 7 weeks F: 2 sessions /Week D: 20 min	60 per session 840 in total	In-season	CMJ, 20 m sprint, COD (Illinois)	E.G.,: All ↑ CG: COD, 20 m Sprint ↑, others ↔	All ↑ in, E.G., vs. CG
Ramirez-Campillo et al. (2020a)	N = 15; TB: 3 years at least, S:M E.G.,: A = 12.9 ± 1.9years, H = 154.0 ± 11.6 cm BM = 44.4 ± 12.5 kg CG: A = 12.6 ± 1.8years, H = 155.9 ± 13.0 cm BM = 45.6 ± 10.3 kg PL: Tier 2 (Trained/Developmental) Maturity metric:NR	PT	Soccer	V-JUMP	L: 8 weeks F: 2 sessions /Week D: 10–15 min	40-70 per session 810 in total	In-season	CMJ, SJ, 30 m sprint, COD (Meylan test)	E.G.,: All ↑ CG: All ↔	All ↑ in, E.G., vs. CG
Vera-Assaoka et al. (2020)	N = 76; TB:3 years at least, S:M E.G.,1: A = 11.2 ± 0.8years, H = 143 ± 5.2 cm BM = 36.8 ± 5.1 kg E.G.,2: A = 14.4 ± 1.0years, H = 163 ± 7.2 cm BM = 54.7 ± 6.6 kg CG1: A = 11.5 ± 0.9years, H = 141 ± 4.0 cm BM = 35.8 ± 3.8 kg CG2: A = 14.5 ± 1.1year, H = 162 ± 8.3 cm BM = 55.8 ± 7.9 kg PL: Tier 2 (Trained/Developmental) Maturity metric:Tanner	PT	Soccer	V-JUMP	L: 7 weeks F: 2 sessions /Week D: 21 min	60 per session 840 in total	In-season	CMJ, 20 m sprint, COD (Illinois)	E.G.,1: CMJ, COD ↑, others ↔ E.G.,2:CMJ, COD ↑,20 m Sprint ↓ CG1: 20 m Sprint ↓, others ↔ CG2: COD, 20 m Sprint ↓, others ↔	20 m Sprint, COD ↑, others ↔ in, E.G.,1 vs. CG1; All ↑ in, E.G.,2 vs. CG2
Ramirez-Campillo et al. (2018)	N = 73; TB: 2 years at least S:M E.G.,1: A = 13.9 ± 1.9 years, H = 153 ± 10 cm, BM = 46.7 ± 10.5 kg E.G.,2: A = 13.1 ± 1.7 years, H = 153 ± 10 cm, BM = 47.2 ± 11.5 kg CG: A = 13.7 ± 1.6 years, H = 155 ± 10 cm, BM = 49.1 ± 11.1 kg PL: Tier 2 (Trained/Developmental) Maturity metric:Tanner	PT	Soccer	V-JUMP	L: 7 weeks F: 2 sessions /Week D: 10–17 min	48-90 per session 906 in total	In-season	CMJ, 20 m sprint, COD (Illinois)	E.G.,1: CMJ, COD, others ↔ E.G.,2: CMJ, COD ↑, others ↔ CG: All ↔	CMJ, COD ↑, others ↔ in, E.G.,1 vs. CG CMJ, COD ↑, others ↔ in, E.G.,2 vs. CG
Gaamouri et al. (2023)	N = 28; TB: 5 years at least, S:FM E.G.,: A = 15.7 ± 0.2years, H = 165 ± 3 cm BM = 63.8 ± 3.3 kg CG: A = 15.8 ± 0.2years, H = 167 ± 3 cm BM = 63.3 ± 4.1 kg PL: Tier 3 (highly trained/National) Maturity metric:Maturity offset/PHV	PT	Handball	MIX	L: 10 weeks F: 2 sessions /Week D: NR	60-90 per session 1,440 in total	In-season	CMJ, SJ, SLJ, COD (T-test)	E.G.,: All ↑ CG: COD ↑, CMJ, SJ, SLJ ↓	All ↑ in, E.G., vs. CG
Noutsos et al. (2021)	N = 33; TB: 2 years at least, S:M E.G.,: A = 12.47 ± 0.2years, H = 155 ± 3 cm BM = 47.7 ± 2.3 kg CG: A = 12.35 ± 0.2years, H = 154 ± 4 cm BM = 48.9 ± 2.8 kg PL: Tier 2 (Trained/Developmental) Maturity metric:Maturity offset/PHV	PT	Handball	MIX	L: 6 weeks F: 2 sessions /Week D: NR	144 per session 1728 in total	In-season	CMJ, SJ,10m, 20 m Sprint, COD (T-test)	E.G.,: COD ↑, others ↔ CG: All ↔	NR
Sankey et al. (2008)	N = 18; TB: NR; S:M A = 14.5 ± 0.5years, H = 174 ± 7 cm BM = 65.2 ± 9.26 kg PL: Tier 3 (highly trained/National) Maturity metric:NR	PT	Rugby	E.G.,1: V-JUMP E.G.,2: MIX	E.G.,1 and, E.G.,2 L: 6 weeks F: 2 sessions /Week D: NR	E.G.,1:110 per session 1,320 in total E.G.,2:80-140 per session 1,320 in total	In-season	CMJ	E.G.,: All ↑ CG: All ↔	NR
Fathi et al. (2019)	N = 40; TB:NR, S:M E.G.,: A = 14.6 ± 0.5years, H = 178.1 ± 4.5 cm BM = 67.9 ± 9.7 kg CG: A = 14.5 ± 0.6years, H = 173.9 ± 7.1 cm BM = 63.4 ± 15.3 kg PL: Tier 2 (Trained/Developmental) Maturity metric:Maturity offset/APHV	PT	Volleyball	V-JUMP	L: 16 weeks F: 1 session /Week D: 35 min	24-50 per session 1,104 in total	Pre-season	CMJ, SJ,5 m,10 m sprint	E.G.,: All ↑ CG: All ↔	All in, E.G., vs. CG ↔

A, age; H, height; BM, body mass; F, female; M, male; TB, training background; PT, plyometric training; NR, not reported; CG, control group; E.G., experimental group; L, length; F, frequency; D, duration; CMJ, countermovement jump; SJ, squat jump; SLJ, standing long jump; COD: agility ability with change of direction; ↑, significantly positive effect (p ≤ 0.05); ↓, significantly negative effect (p ≤ 0.05); ↔, no effect (p > 0.05),PL: athlete level as originally reported/defined in the included studies; McKay tier, athlete caliber classified using the McKay Participant Classification Framework (PCF; Tier 0–5).

#### 3.2.1 Sample size

Thirty-one articles included a total of 1,033 participants (127 females, 906 males), with individual studies ranging from 15 to 76 participants. This comprised 247 basketball players, 667 footballers, 61 handball players, 40 volleyball players, and 18 rugby players.

#### 3.2.2 Sex

Twenty-five studies included male participants, five studies included female participants, and one study included both.

#### 3.2.3 Biological maturity

Eight studies used maturity offset, seven used Tanner staging, and sixteen did not report maturity-related information.

#### 3.2.4 Playing level

Based on the McKay Participant Classification Framework, most included studies involved developmental-level athletes, while a smaller number examined national-level players.

#### 3.2.5 Training duration

The studies ranged in duration from 6 to 12 weeks, with only one lasting 16 weeks.

#### 3.2.6 Training frequency

Twenty-eight studies employed twice-weekly training. Two studies employed a once-weekly frequency. Only one study reported a frequency of three times a week.

#### 3.2.7 Session duration

Twenty-four studies indicated single-session lengths varying from 15 to 60 min.

#### 3.2.8 Training volume (total number of jumps)

The number of jumps in a single session was between 24 and 220. The total number of jumps ranged from 512 to 2,880.

#### 3.2.9 Intervention methods

Twenty studies combined horizontal and vertical PT. Eight studies employed vertical PT. Two studies included only horizontal PT. One study reported both vertical and horizontal PT.

#### 3.2.10 Seasonal training timing

Twenty-three studies reported implementing training programs during the season, while six studies reported pre-season implementation. Three studies did not report this information.

### 3.3 Risk of bias assessment and certainty of evidence

**FIGURE 2:**
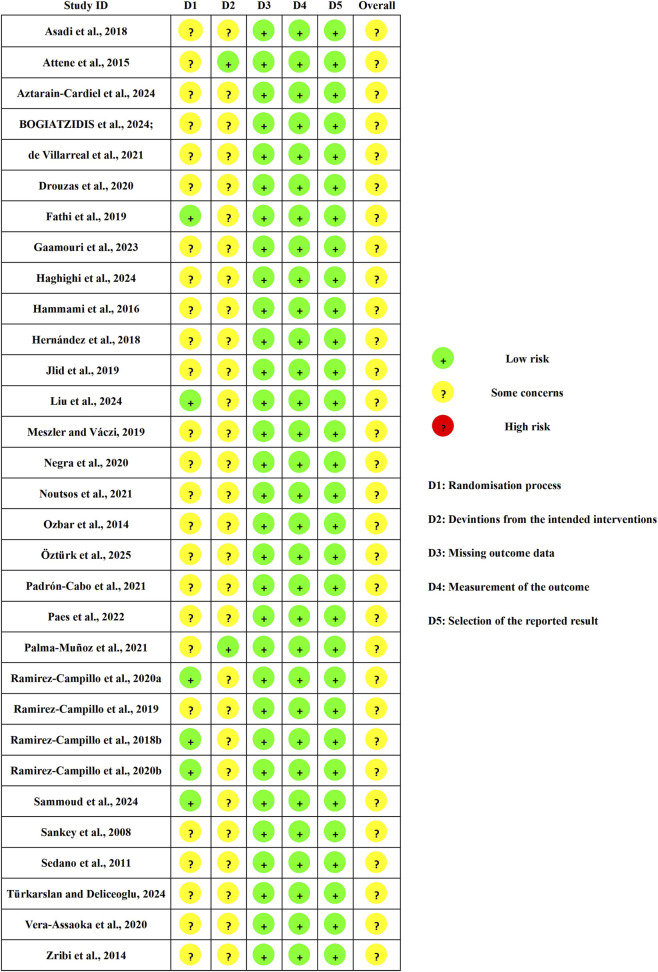
Risk of overall bias.

**FIGURE 3:**
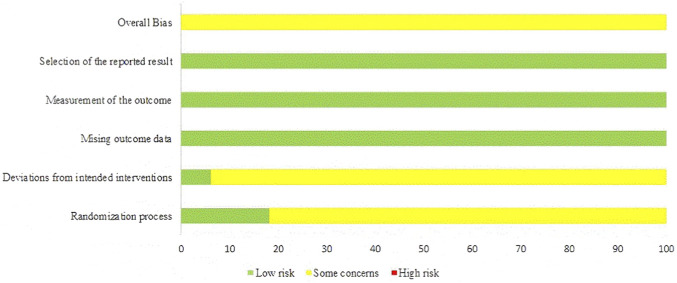
RoB-2 assessments.

Detailed results of the bias risk assessment for each area and overall are presented in Figures 2, 3. The primary sources of risk of bias were “randomization process” and “deviation from the intended intervention”, as it is challenging to blind participants and assessors in sports training. Only six studies explicitly described the randomization process (Hernández et al., 2018; Fathi et al., 2019; Ramirez-Campillo et al., 2020a; Ramirez-Campillo et al., 2020b; Liu et al., 2024; Sammoud et al., 2024). Only two studies were rated as having a low risk of bias in the domain of deviations from the intended interventions (Attene et al., 2015; Palma-Muñoz et al., 2021). For the primary outcome, the GRADE evidence quality level is low or very low (see Table 3). Downgrading was mainly due to risk of bias, inconsistency, and imprecision, with suspected publication bias for several outcomes.

**TABLE 3:** GRADE analyses.

Quality assessment							
Number of studies (Participants)	Study design	Risk of bias	Inconsistency	Indirectness	Imprecision	Other considerations	Quality
Countermovement jump							
27 (931)	RCT	Serious^a^	serious^b^	No	serious^c^	Publication bias	⨁◯◯◯ Very low
Squat jump							
14 (502)	RCT	Serious^a^	NO	NO	serious^c^	NO	⨁⨁◯◯ low
Countermovement jump with arms							
4 (115)	RCT	Serious^a^	serious^b^	NO	serious^c^	Publication bias	⨁◯◯◯ Very low
Standing long jump							
8 (240)	RCT	Serious^a^	serious^b^	NO	serious^c^	Publication bias	⨁◯◯◯ Very low
≤10-m linear sprint							
10 (484)	RCT	Serious^a^	serious^b^	NO	serious^c^	NO	⨁⨁◯◯ low
20-m linear sprint							
16 (602)	RCT	Serious^a^	NO	NO	serious^c^	NO	⨁⨁◯◯ low
30-m linear sprint							
5 (112)	RCT	Serious^a^	serious^b^	NO	serious^c^	Publication bias	⨁◯◯◯ Very low
Change-of-direction							
19 (632)	RCT	Serious^a^	serious^b^	NO	serious^c^	Publication bias	⨁◯◯◯ Very low

^a^ Some included studies had methodological limitations.

^b^ There was significant heterogeneity in the effect sizes observed across studies.

^c^ The confidence intervals for the effect estimates for multiple outcome indicators were too wide, and the total sample size for some outcomes was lower than that required to reliably detect the true effect. Publication bias: The Egger’s test result was significant (P < 0.05).

### 3.4 Meta-analysis results

The results of the eight meta-analyses are presented in Table 4, and the corresponding forest plots are provided in Figures 4–11.

**TABLE 4:** Synthesis of results across included studies regarding the effects of plyometric training on physical fitness measures.

Fitness attribute	n^a^	ES (95%CI)	p (Overall effect)	p (Heterogeneity)	I^2^ (%)	RW (%)	Egger’test (p)
Countermovement jump	27,40,30,931	0.89 (0.59–1.19)	<0.001	<0.001	75	0.5–3.2	0.001
Countermovement jump with arms	4,54,115	1.00 (0.14–1.86)	= 0.02	0.004	74	16.1–23.3	NR
Squat jump	14,19,14,502	0.48 (0.22–0.75)	<0.001	0.01	48	3.7–7.4	0.636
Standing long jump	8,13,10,240	1.10 (0.62–1.58)	<0.001	0.002	62	5.7–9.4	NR
≤10-m linear sprint	10,19,14,484	−0.59 (−0.87 to −0.32)	<0.001	0.009	49	2.3–7.2	0.215
20-M linear sprint	16,25,20,602	−0.42 (−0.63 to −0.21)	<0.001	0.06	33	1.6–6.0	0.415
30-M linear sprint	5,75,112	−0.97 (−1.68 to −0.26)	0.008	0.02	59	8.3–20.1	NR
Change-of-direction	19,28,21,632	−0.73 (−1.02 to −0.45)	<0.001	<0.001	60	1.3–4.9	0.007

n^a^ Data denote the number of studies that provided data for the analysis, the number of experimental groups, the number of control groups, and the total number of adolescent team sport players included in the analysis, respectively. NR, Less than 10 studies were included and publication bias was not evaluated.

**FIGURE 4:**
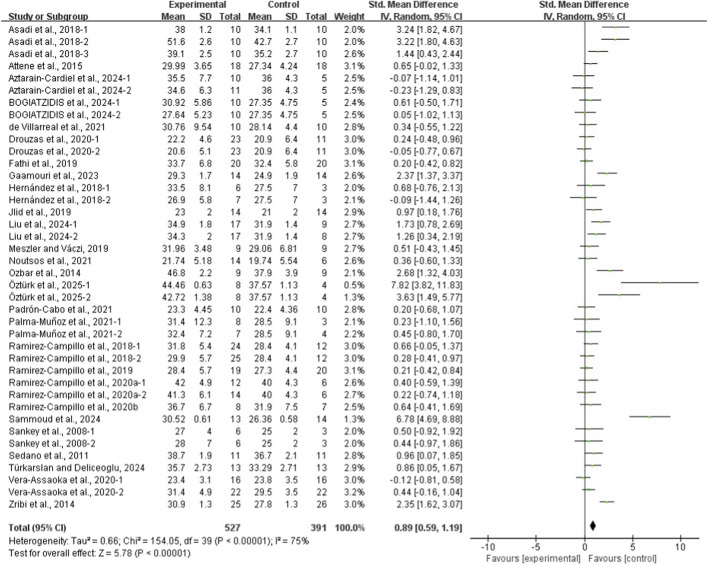
Forest plot of the effect of plyometric training on CMJ in adolescent team-sport athletes.

**FIGURE 5:**
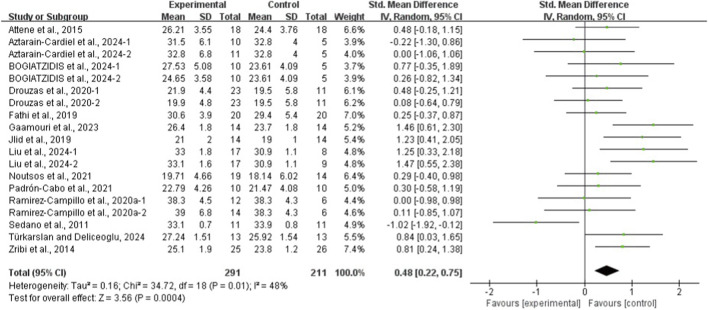
Forest plot of the effect of plyometric training on SJ in adolescent team-sport athletes.

**FIGURE 6:**
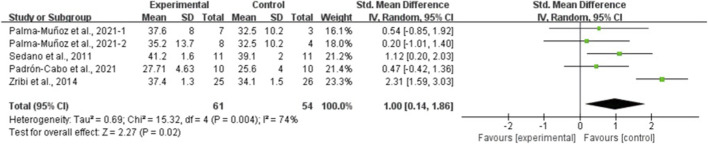
Forest plot of the effect of plyometric training on CMJA in adolescent team-sport athletes.

**FIGURE 7:**
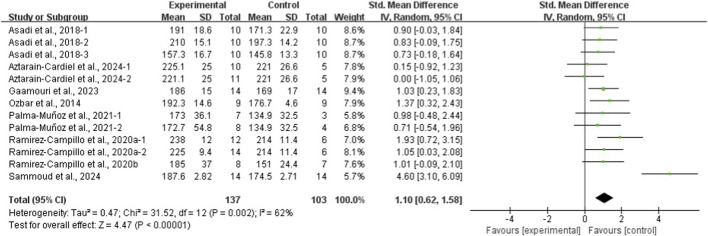
Forest plot of the effect of plyometric training on SLJ in adolescent team-sport athletes.

**FIGURE 8:**
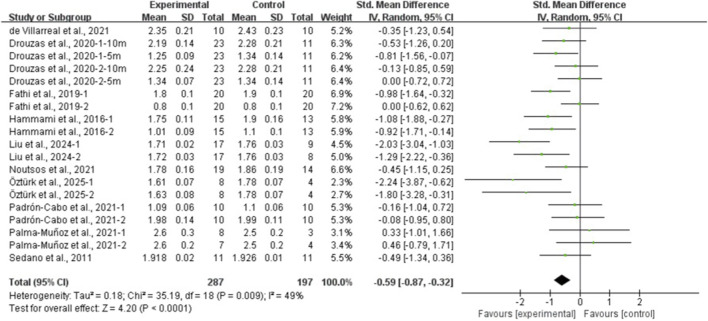
Forest plot of the effect of plyometric training on ≤10-m linear sprint in adolescent team-sport athletes.

**FIGURE 9:**
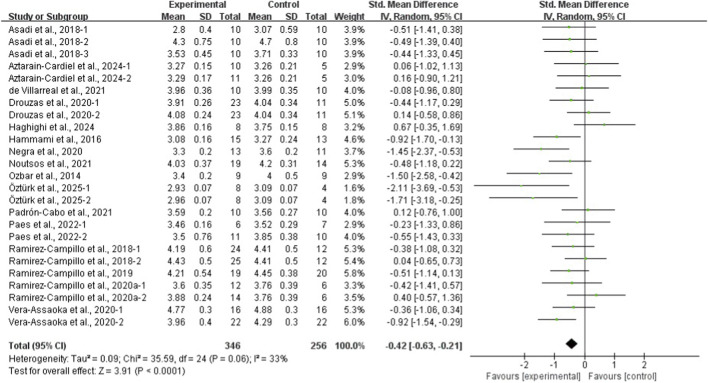
Forest plot of the effect of plyometric training on 20-m linear sprint in adolescent team-sport athletes.

**FIGURE 10:**
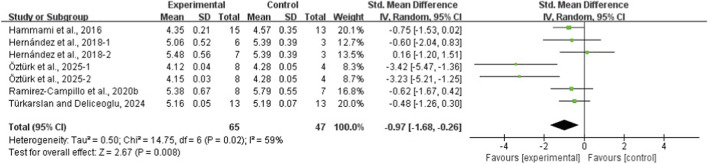
Forest plot of the effect of plyometric training on 30-m linear sprint in adolescent team-sport athletes.

**FIGURE 11:**
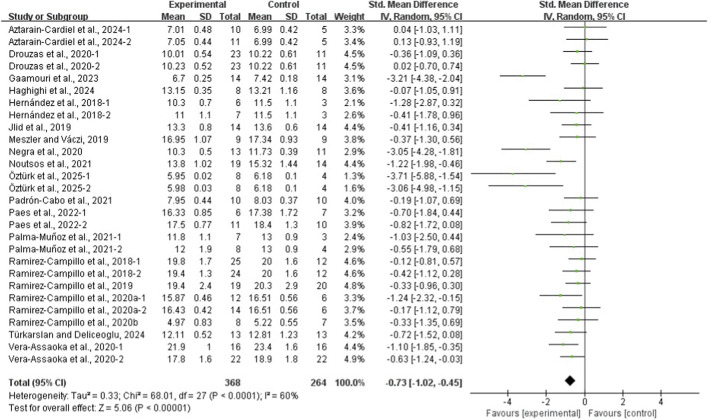
Forest plot of the effect of plyometric training on COD in adolescent team-sport athletes.

PT significantly improved jump performance (CMJ: ES = 0.89, 95% CI: 0.59–1.19, I^2^ = 75%; CMJA: ES = 1.00, 95% CI: 0.14–1.86, I^2^ = 74%; SJ: ES = 0.48, 95% CI: 0.22–0.75, I^2^ = 48%; SLJ: ES = 1.10, 95% CI: 0.62–1.58, I^2^ = 62%). For time-based outcomes, PT also improved linear sprint performance (≤10 m: ES = −0.59, 95% CI: −0.87 to −0.32, I^2^ = 49%; 20 m: ES = −0.42, 95% CI: −0.63 to −0.21, I^2^ = 33%; 30 m: ES = −0.97, 95% CI: −1.68 to −0.26, I^2^ = 59%) and COD (ES = −0.73, 95% CI: −1.02 to −0.45, I^2^ = 60%).

### 3.5 Additional analyses

A total of 17 subgroup analyses were conducted (Table 5). For CMJ, significant between-subgroup differences were observed for age (p = 0.04) and training duration (p = 0.009), with the largest improvements in athletes aged 16–18.99 years and in programmes lasting ≥8 to <10 weeks. For SJ, significant between-subgroup differences were observed for training duration (p < 0.001), with the largest improvement in programmes lasting ≥8 to <10 weeks. Similarly, for the ≤10-m and 20-m linear sprints, training duration showed significant between-subgroup differences (p < 0.001), with the greatest improvements in programmes lasting ≥8 to <10 weeks. Detailed information on the pooled effect size, heterogeneity, and the number of included studies for each subgroup can be found in Table 5.

**TABLE 5:** Meta-subgroup analysis results.

Subgroup	Type	K	n	ES (95%CI)	p (Overall effect)	I^2^ (%)	p (Heterogeneity)	p (Subgroup difference)
Countermovement jump								
Age	10–12.99	7	220	0.48 (−0.01–0.97)	0.06	64	0.005	0.03
	13–15.99	17	538	0.76 (0.38–1.14)	<0.001	74	<0.001	
	16–18.99	6	173	1.83 (0.98–2.67)	<0.001	77	<0.001	
Sex	M	23	831	0.82 (0.51–1.13)	<0.001	74	<0.001	0.24
	FM	4	100	1.47 (0.42–2.52)	0.006	79	0.002	
Training duration	≤7	16	558	0.68 (0.34–1.02)	<0.001	69	<0.001	0.009
	≥8 < 10	6	195	1.92 (1.15–2.70)	<0.001	73	<0.001	
	≥10	5	220	0.56 (0.03–1.08)	0.04	70	0.002	
Total number of jumps	≤800	5	158	1.32 (0.51–2.13)	0.001	78	<0.001	0.45
	>800 ≤ 1,400	14	430	0.85 (0.39–1.32)	<0.001	76	<0.001	
	>1,400	10	343	0.73 (0.30–1.16)	0.001	69	<0.001	
Squat jump								
Age	10–12.99	5	200	0.54 (0.22–0.86)	0.001	16	0.31	0.76
	13–15.99	6	244	0.51 (0.16–0.86)	0.004	24	0.24	
	16–18.99	3	111	0.36 (−0.55–1.27)	0.44	79	<0.001	
Training duration	≤7	6	185	0.35 (0.05–0.65)	0.02	0	0.44	<0.001
	≥8 < 10	4	156	1.08 (0.73–1.42)	<0.001	0	0.60	
	≥10	4	160	0.13 (−0.32–0.57)	0.58	42	0.12	
Total number of jumps	≤800	3	86	0.73 (−0.02–1.47)	0.06	60	0.06	0.61
	>800 ≤ 1,400	3	92	0.30 (−0.11–0.72)	0.15	24	0.73	
	>1,400	9	324	0.46 (0.10–0.82)	0.01	58	0.01	
≤10-m linear sprint								
Training duration	≤7	4	115	−0.17 (−0.54 to 0.21)	0.38	0	0.80	<0.001
	≥8 < 10	3	131	−1.35 (−1.76 to −0.95)	<0.001	0	0.43	
	≥10	3	238	−0.41 (−0.71 to −0.11)	0.009	21	0.27	
Total number of jumps	≤800	4	158	−0.78 (−1.33 to −0.24)	0.005	68	0.02	0.42
	>800 ≤ 1,400	4	135	−0.67 (−1.33 to 0.00)	0.05	63	0.01	
	>1,400	3	191	−0.39 (−0.69 to −0.09)	0.01	0	0.68	
20-M linear sprint								
Age	10–12.99	6	197	−0.39 (−0.75 to −0.04)	0.03	30	0.20	0.44
	13–15.99	9	305	−0.34 (-0.60 to −0.09)	0.008	13	0.32	
	16–18.99	4	100	−0.85 (−1.57 to −0.13)	0.02	60	0.03	
Sex	M	14	547	−0.42 (−0.62 to −0.21)	<0.001	23	0.16	0.95
	FM	3	55	−0.45 (−1.62 to 0.71)	0.45	76	0.02	
Training duration	≤7	11	440	−0.29 (−0.49 to −0.10)	0.003	0	0.66	<0.001
	≥8 < 10	4	94	−1.35 (−1.82 to −0.88)	<0.001	0	0.66	
Total number of jumps	≤800	5	147	−0.54 (−0.94 to −0.15)	0.007	26	0.23	0.37
	>800 ≤ 1,400	9	316	−0.47 (−0.80 to −0.14)	0.005	45	0.04	
	>1,400	3	139	−0.19 (−0.55 to 0.16)	0.29	0	0.47	
Change-of-direction								
Age	10–12.99	7	224	−0.85 (−1.50 to −0.19)	0.01	71	0.002	0.24
	13–15.99	11	346	−0.58 (−0.88 to −0.28)	<0.001	48	0.01	
	16–18.99	2	62	−1.80 (−3.28 to −0.32)	0.02	77	0.004	
Sex	M	16	537	−0.66 (−0.94 to −0.39)	<0.001	51	<0.001	0.82
	FM	4	83	−1.08 (−2.32 to 0.16)	0.09	84	<0.001	
Training duration	≤7	13	447	−0.52 (−0.71 to −0.32)	<0.001	0	0.69	0.08
	≥8 < 10	4	89	−1.98 (−3.29 to −0.66)	0.003	81	<0.001	
	≥10	2	96	−1.12 (−2.74 to 0.51)	0.18	91	<0.001	
Total number of jumps	≤800	4	69	−1.02 (−2.37 to 0.34)	0.14	82	<0.001	0.63
	>800 ≤ 1,400	10	323	−0.58 (−0.90 to −0.26)	<0.001	41	0.05	
	>1,400	7	240	−0.83 (−1.34 to −0.32)	0.002	67	0.001	

K, the number of studies that provided data for the analysis. n, the total number of adolescent team sport players included in the analysis.

When there were at least 10 studies for the same outcome measure, age, training duration, and training volume (total number of jumps) were used as covariates in a meta-regression analysis (see Table 6). The results indicated that age was significantly associated with improvements in CMJ (β = 0.211, p = 0.026), ≤10-m linear sprint (β = −0.119, p = 0.031), 20-m linear sprint (β = −0.117, p = 0.023), and COD (β = −0.163, p = 0.048). Training duration was significantly associated with improvements in 20-m linear sprint time (β = −0.206, p = 0.034). The total number of jumps was significantly associated with improvements in the SJ, although the magnitude of the association was small (β = −0.00048, p = 0.049).

**TABLE 6:** Multivariate meta-regression for training variables to predict plyometric training effects.

Covariate	Coefficient	95%CI	95%CI	t	p
Countermovement jump height					
Intercept	−1.41632	−4.322483	1.489842	−0.99	0.330
Training duration	−0.0329261	−0.2196788	0.1538266	−0.36	0.723
Total ground contacts	−0.0003162	−0.0011059	0.0004735	−0.81	0.422
Age	0.2112292	0.0266013	0.395857	2.32	0.026*
Squat jump					
Intercept	1.35279	−0.6153355	3.320915	1.47	0.164
Training duration	−0.0105397	−0.1121702	0.0910909	−0.22	0.828
Total ground contacts	−0.0004846	−0.0009667	−2.52e-06	−2.14	0.049*
Age	−0.0069975	−0.1308152	0.1168202	1.47	0.164
≤10-m linear sprint					
Intercept	0.8048927	−0.9484814	2.558267	0.98	0.343
Training duration	−0.004712	−0.0968085	0.0873846	−0.11	0.915
Total ground contacts	0.0002311	−0.0002817	0.000744	0.96	0.352
Age	−0.1186683	−0.2316182	−0.0123101	−2.38	0.031*
20-M linear sprint					
Intercept	2.183454	0.0028397	4.364067	2.08	0.050
Training duration	−0.206024	−0.3948244	−0.0172236	−2.27	0.034*
Total ground contacts	0.0004498	−0.0000189	0.0009184	2.00	0.059
Age	−0.1173318	−0.2165343	−0.0181293	−2.46	0.023*
Change-of-direction					
Intercept	3.288426	0.0237177	6.553134	2.08	0.048
Training duration	−0.2815789	−0.562539	−0.0006188	−2.07	0.050
Total ground contacts	0.0001669	−0.0005559	0.0008897	0.48	0.638
Age	−0.1625591	−0.3235699	−0.0015483	−2.08	0.048*

*p < 0.05.

### 3.6 Publication bias and sensitivity analyses

Publication bias was assessed only for outcomes with ≥10 studies. Therefore, Egger tests were performed for five outcomes (see Table 4). CMJ and COD showed a risk of publication bias. By using the trim-and-fill method for adjustment, the results' significance remained unchanged, indicating that publication bias did not significantly affect the effect size.

Sensitivity analyses indicated that the pooled ES was robust for CMJ, SJ, SLJ, all sprint outcomes, and COD, whereas the pooled ES for CMJA was sensitive to omission of individual studies (Supplementary Figures S1–S8).

## 4 Discussion

Overall, this systematic review and meta-analysis indicates that plyometric training (PT) can enhance the jumping, linear sprint, and change of direction (COD) performance of adolescent team sport athletes. However, there is moderate to high heterogeneity in multiple outcome measures, and the certainty of evidence is generally low to very low. Therefore, the results of subgroup analysis and meta-regression should be interpreted with caution and regarded as exploratory findings, which are not sufficient to form definitive conclusions.

### 4.1 Jump performance

The meta-analysis showed that PT can effectively improve jump performance in youth team-sport athletes. Specifically, PT significantly improved CMJ (ES = 0.89), CMJA (ES = 1.00), and SLJ (ES = 1.10), whereas the improvement in SJ (ES = 0.48) was smaller but still statistically significant. CMJA results should be interpreted cautiously because they were not robust in sensitivity analyses.

Notably, the improvement in CMJ was clearly larger than that in SJ, suggesting that PT may be more sensitive for jumps involving a countermovement. This may be related to the relatively long pause at the bottom position of the SJ (three to five s). Such a pause may reduce the use of elastic energy stored during the eccentric phase, forcing the movement to rely mainly on concentric contraction, and thereby limiting the contribution of the SSC (MacDougall and Sale, 2014; Stojanović et al., 2017). Improvements in jump performance following PT may be related to structural and neuromuscular adaptations, such as muscle fiber hypertrophy and improved tendon collagen properties, which increase tendon stiffness (Pääsuke et al., 2001; Shepstone et al., 2005), enhance the rapid recruitment of high-threshold motor units, improve central nervous system excitability and reflex control, and strengthen intermuscular and intramuscular coordination (Markovic and Mikulic, 2010; Seiberl et al., 2021).

Regarding potential moderators, subgroup and meta-regression analyses suggested that age and training duration may be associated with the CMJ training effect. The subgroup aged 16–18.99 years showed a greater improvement in CMJ performance (ES = 1.83, p = 0.04), and meta-regression similarly demonstrated a significant association between age and CMJ improvement (β = 0.211, p = 0.026). Importantly, the subgroup and meta-regression results were consistent, suggesting that age may be associated with PT responsiveness. A reasonable explanation is that in older adolescent athletes, a more mature central nervous system and higher levels of testosterone and growth hormone may promote structural and neuromuscular adaptations (Moran J. J. et al., 2017; Radnor et al., 2018; Ramirez-Campillo et al., 2023). In contrast, in younger athletes, lower hormone levels may limit structural adaptations, and their improvements are more related to neuromuscular optimization (Tumkur Anil Kumar et al., 2021). However, because age was used as a proxy rather than directly assessed biological maturity, this interpretation should be considered cautiously.

For training duration, CMJ showed a larger improvement in programmes lasting ≥8 to <10 weeks (ES = 1.92, p = 0.009), and the SJ duration subgroup showed a similar trend (ES = 1.08, p < 0.001). Taken together, the current evidence indicates that programmes lasting ≥8 to <10 weeks may yield clearer improvements in jump performance, without showing that “longer is always better.” The meta-regression indicated a very small negative correlation between the total number of jumps and SJ improvement (β = −0.00048, p = 0.049), which has limited practical significance and should be interpreted with caution. However, the current evidence is insufficient to support the idea that increasing the total number of jumps leads to better training outcomes. One possible explanation is that, because the neuromuscular system of adolescents is still developing, excessive training volume may lead to central nervous system fatigue and high energy expenditure, resulting in impaired neural regulation and the accumulation of metabolic stress. These factors may compromise explosive performance and increase the risk of sports-related injuries (Cairns, 2006; Schoenfeld, 2010; Enoka and Duchateau, 2016). In contrast, appropriately prescribed training duration and volume may facilitate the restoration of energy reserves and muscle tissue repair following high-intensity training, thereby promoting supercompensation (Şahin et al., 2022; Luo et al., 2025). Notably, rapid growth around peak height velocity (PHV) may be associated with a further increase in injury risk (Faigenbaum et al., 2009; Luke et al., 2011). Therefore, greater emphasis should be placed on balancing training load and recovery during this stage.

### 4.2 Linear sprinting

The meta-analysis showed that plyometric training (PT) can significantly improve linear sprint performance in adolescent team-sport athletes. PT significantly improved ≤10-m (ES = −0.59), 20-m (ES = −0.42), and 30-m sprint performance (ES = −0.97). In practical terms, adding PT to regular sport-specific training may benefit both short-distance acceleration and longer-distance sprint performance. PT may enhance sprint performance through improved neural drive and neuromuscular coordination, increased lower-limb stiffness, and improved rapid force production, which together may reduce ground contact time and increase step frequency (Ross et al., 2001; Mackala and Fostiak, 2015; Tomalka et al., 2020).

Regarding potential moderators, subgroup analysis indicated that for ≤10-m sprint, programmes lasting ≥8 to <10 weeks produced larger improvements (ES = −1.35), with significant between-subgroup differences compared with ≤7 weeks and ≥10 weeks (p < 0.001). A similar pattern was also observed for 20 m sprint (ES = −1.35, p < 0.001). These findings suggest that a duration of ≥8 to <10 weeks may be more favorable for sprint improvements, but this should not be interpreted as a definitive “optimal” training duration. A reasonable explanation is that shorter programmes may provide insufficient accumulated stimulus, whereas longer programmes may involve excessive SSC loading, which may reduce tendon stiffness and make muscle fatigue and a neuromuscular adaptation plateau more likely, thereby compromising recovery (Komi, 2000; Nicol et al., 2006).

For age-related effects, meta-regression showed that age was associated with the training effect for both ≤10-m and 20-m sprints (≤10-m: β = −0.118, p = 0.031; 20-m: β = −0.117, p = 0.023), suggesting that older athletes may achieve larger improvements. With growth and development, progressive central nervous system maturation and hormonal changes may facilitate structural and neuromuscular adaptations (Lloyd and Oliver, 2013; Radnor et al., 2018; Silva et al., 2022); meanwhile, increases in lower-limb length may optimize the stride length–frequency combination and translate more effectively into sprint performance (Asadi et al., 2018). In addition, older athletes typically have longer trained experience, more stable neuromuscular control, and more consistent sprint technique, which may facilitate the translation of PT stimuli into sprint gains (Fort-Vanmeerhaeghe et al., 2016). In terms of the total number of jumps, the current evidence is insufficient to support the claim that simply increasing the total number of jumps can lead to better sprint training results. Further research is needed to verify this.

### 4.3 Agility (change-of-direction)

The meta-analysis demonstrated that plyometric training (PT) significantly improves change-of-direction (COD) performance in adolescent team-sport athletes, with a moderate overall effect size (ES = −0.73). Good COD is essential in team sports such as basketball, football, and handball, as it is closely related to offensive and defensive actions, the creation of scoring opportunities, and may be associated with reduced injury risk. These findings indicate that incorporating PT into regular sport-specific training may enhance COD and contribute to improved overall sport performance.

During COD tasks, athletes are required to rapidly transition between deceleration and re-acceleration, a process that fundamentally relies on eccentric–concentric muscle actions and the stretch–shortening cycle (SSC) of the lower-limb musculature (Sheppard and Young, 2006; Chaabene et al., 2018). PT imposes substantial inertial and eccentric braking loads during the deceleration phase, which may enhance eccentric strength and neural drive, improve inter- and intramuscular coordination, increase SSC efficiency, and enhance balance and joint stability (Markovic and Mikulic, 2010; Granacher et al., 2015; Chaabene et al., 2018; Jimenez-Iglesias et al., 2024). Collectively, these adaptations may facilitate faster deceleration control and more efficient re-acceleration, ultimately leading to improvements in COD performance.

Regarding potential moderators, age-based subgroup analyses did not reach statistical significance; however, meta-regression indicated that age may be associated with the COD training effect (β = −0.163, p = 0.048), suggesting that training responses may differ across age groups. With respect to training duration, no significant differences were observed between subgroups, although meta-regression revealed a borderline trend (β = −0.282, p = 0.050). In terms of the total number of jumps, the current evidence does not support achieving greater improvements in COD performance simply by increasing the total number of jumps.

### 4.4 Study limitations

Several limitations of this study should be considered when interpreting the results. The risk of bias assessment revealed that most included studies were assessed to have “some concerns” or a “high risk” of bias, primarily in the “randomization process” and “deviation from the intended interventions”, as it is challenging to blind participants and assessors in sports training. Furthermore, the GRADE quality of evidence indicated that the quality of evidence for the outcome indicators was mainly low to very low. These limitations may bias estimates of the impact of PT on the physical fitness of adolescent team athletes. Only five of the included studies focused on female adolescents, limiting the applicability of the findings to female adolescent sports teams. Therefore, more research on female adolescents is needed in the future. The information on biological maturity was insufficient and inconsistent (8 articles for PHV, seven articles for Tanner, and 16 articles did not report), and the types of maturity indicators were not uniform, which limited the further examination of the differences in the maturity stages. Although this study used the WHO age-based developmental stages for age grouping, this grouping does not represent the directly measured biological maturity and may mask the impact of true maturity differences on training adaptation. Finally, the information on ground contact time was insufficiently reported: most studies did not provide quantifiable ground contact time data or unified monitoring methods, and some only made qualitative descriptions such as “quick landing”. Safety reporting was a major limitation of the evidence base. As adverse events were rarely reported, safety outcomes could not be synthesized, and PT safety remains uncertain in this population.

### 4.5 Practical applications

PT is a feasible and effective training method for enhancing the jumping, sprinting and COD abilities of youth team sports athletes. A training program conducted twice a week for a duration of ≥8 weeks to <10 weeks can lead to more stable improvements in multiple physical performance indicators. In practical training, the focus of the training should not merely be on achieving a high total number of jumps, but should be adjusted individually based on the athlete’s developmental stage, training experience, recovery status and season workload.

## 5 Conclusion

Plyometric training improves jumping, linear sprint, and change-of-direction performance in adolescent team-sport athletes. Age may moderate the training response, with athletes aged 16–18.99 years showing larger improvements in CMJ, ≤10-m linear sprint, 20-m linear sprint and COD. Interventions lasting ≥8 to <10 weeks were associated with more consistent gains, particularly for CMJ, ≤10-m linear sprint, and 20-m linear sprint. The available evidence does not indicate that simply increasing total number of jumps is consistently associated with greater performance gains.

## Data Availability

The original contributions presented in the study are included in the article/Supplementary Material, further inquiries can be directed to the corresponding author.
