# Supplementary figures and images for: The effects of plyometric training on physical fitness in adolescent team sports: a systematic review and meta-analysis
Source: Front Physiol. 2026 Jan 21;17:1760239. doi: 10.3389/fphys.2026.1760239 (PMC12867812; doi:10.3389/fphys.2026.1760239)

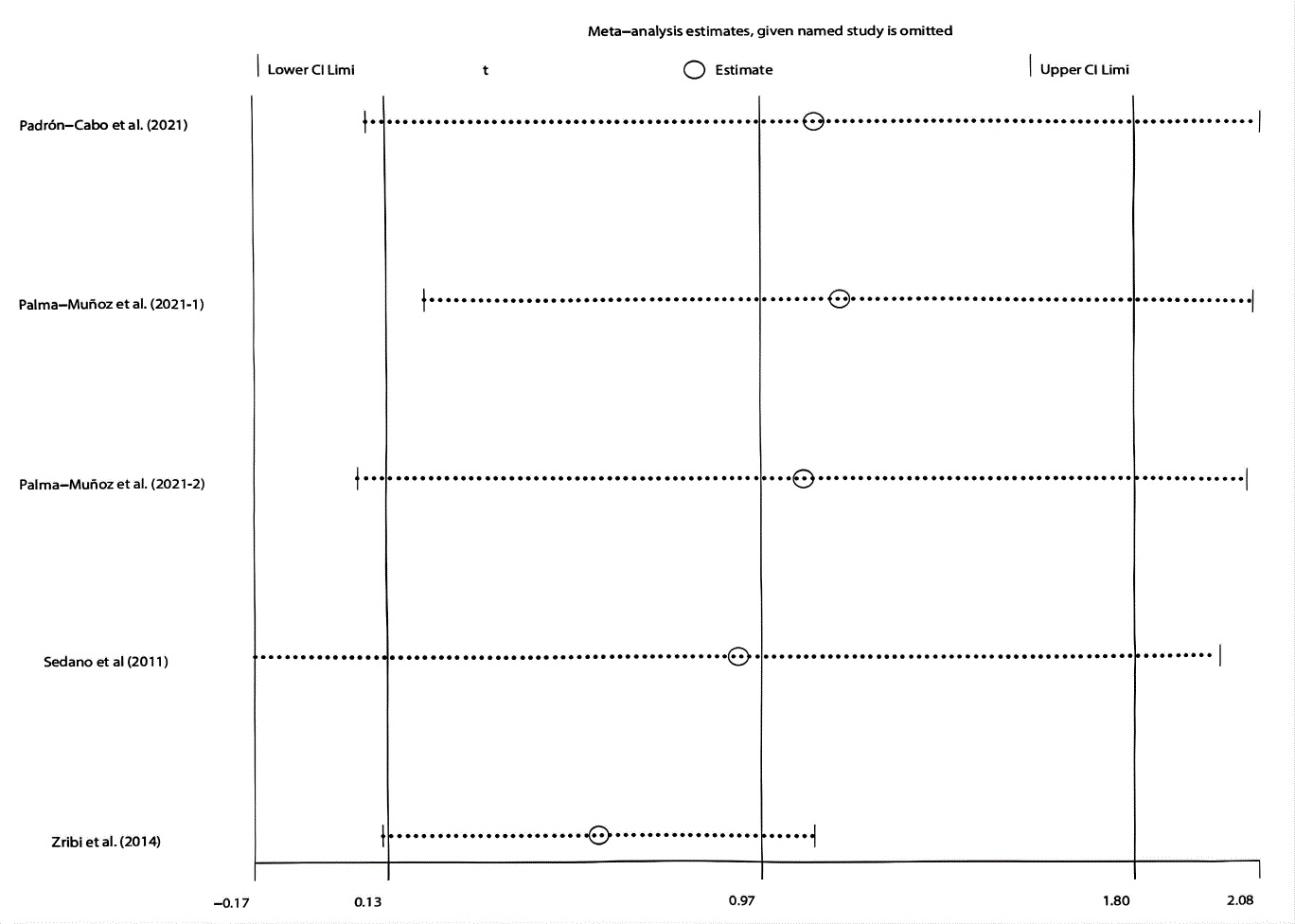

Supplement: Supplementary file 1 [file Image3.jpeg]

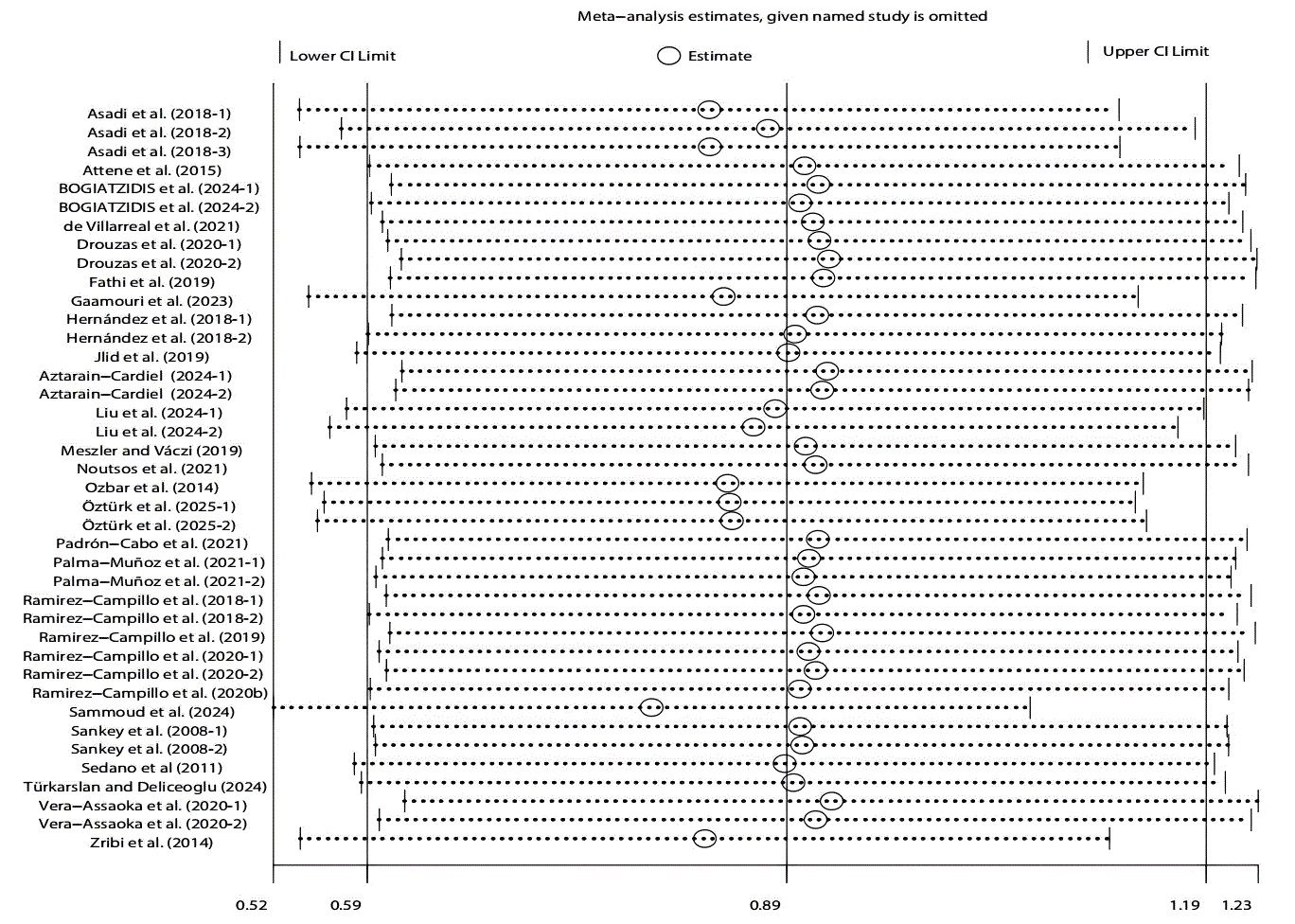

Supplement: Supplementary file 2 [file Image1.jpeg]

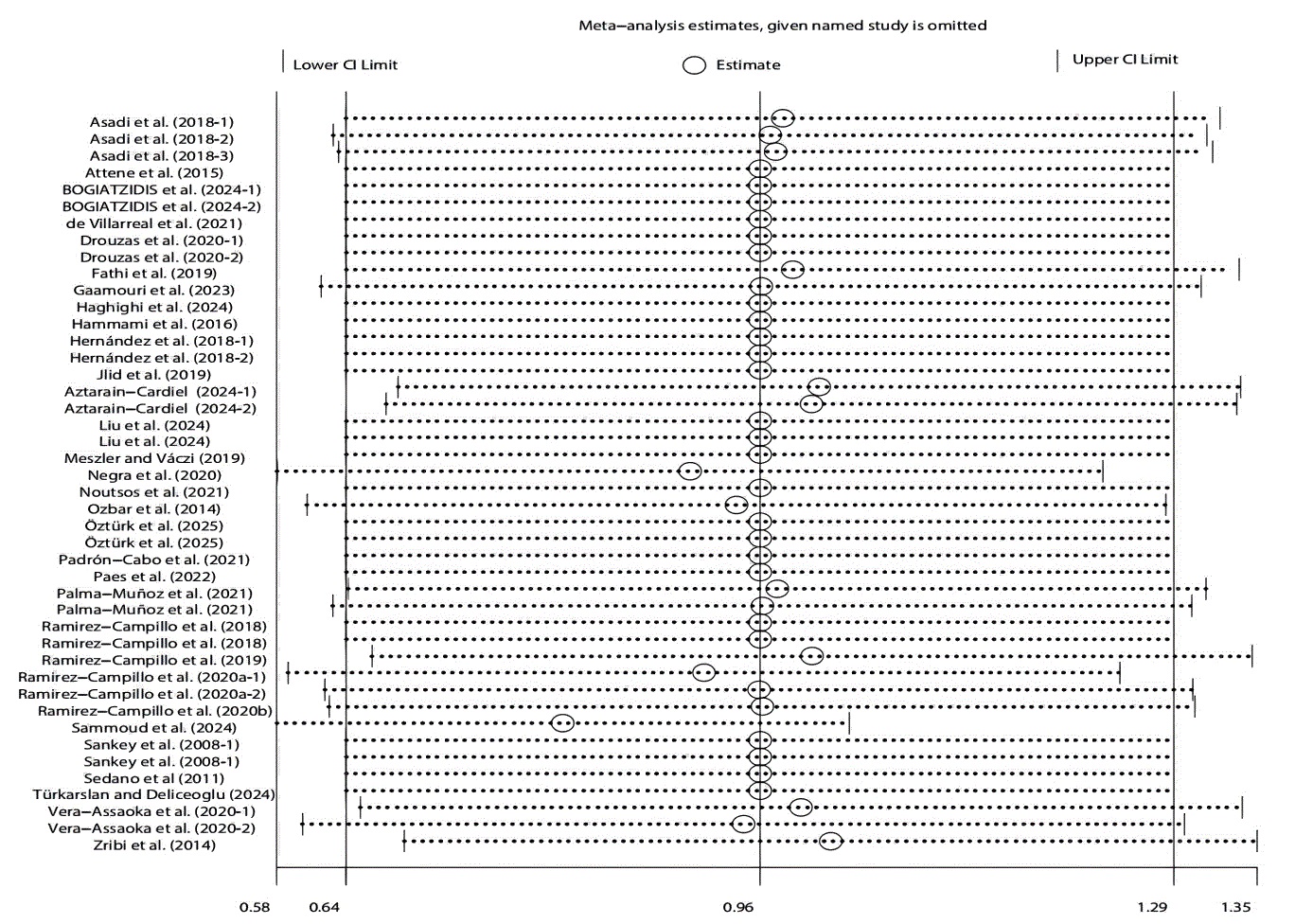

Supplement: Supplementary file 3 [file Image4.jpeg]

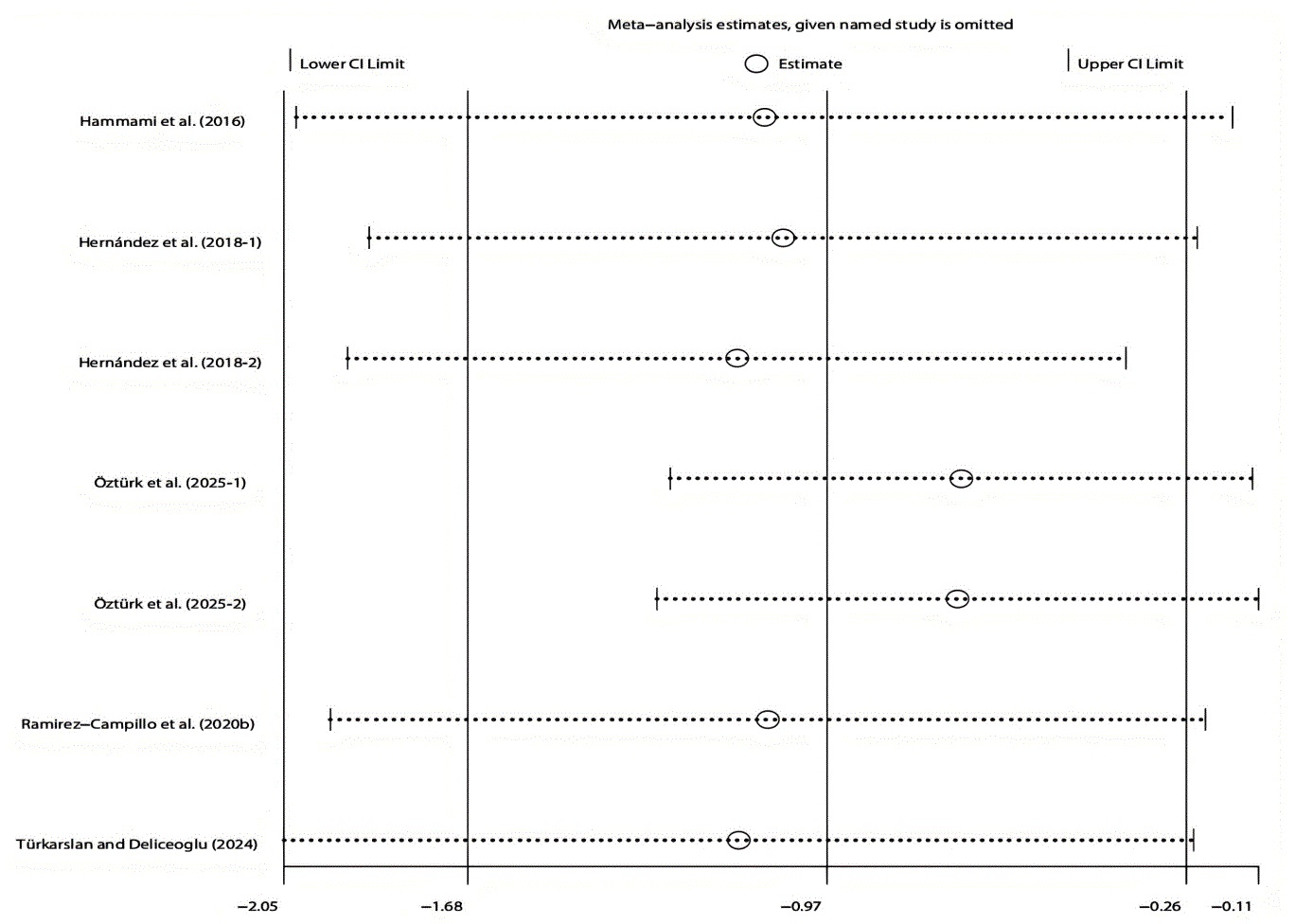

Supplement: Supplementary file 4 [file Image7.jpeg]

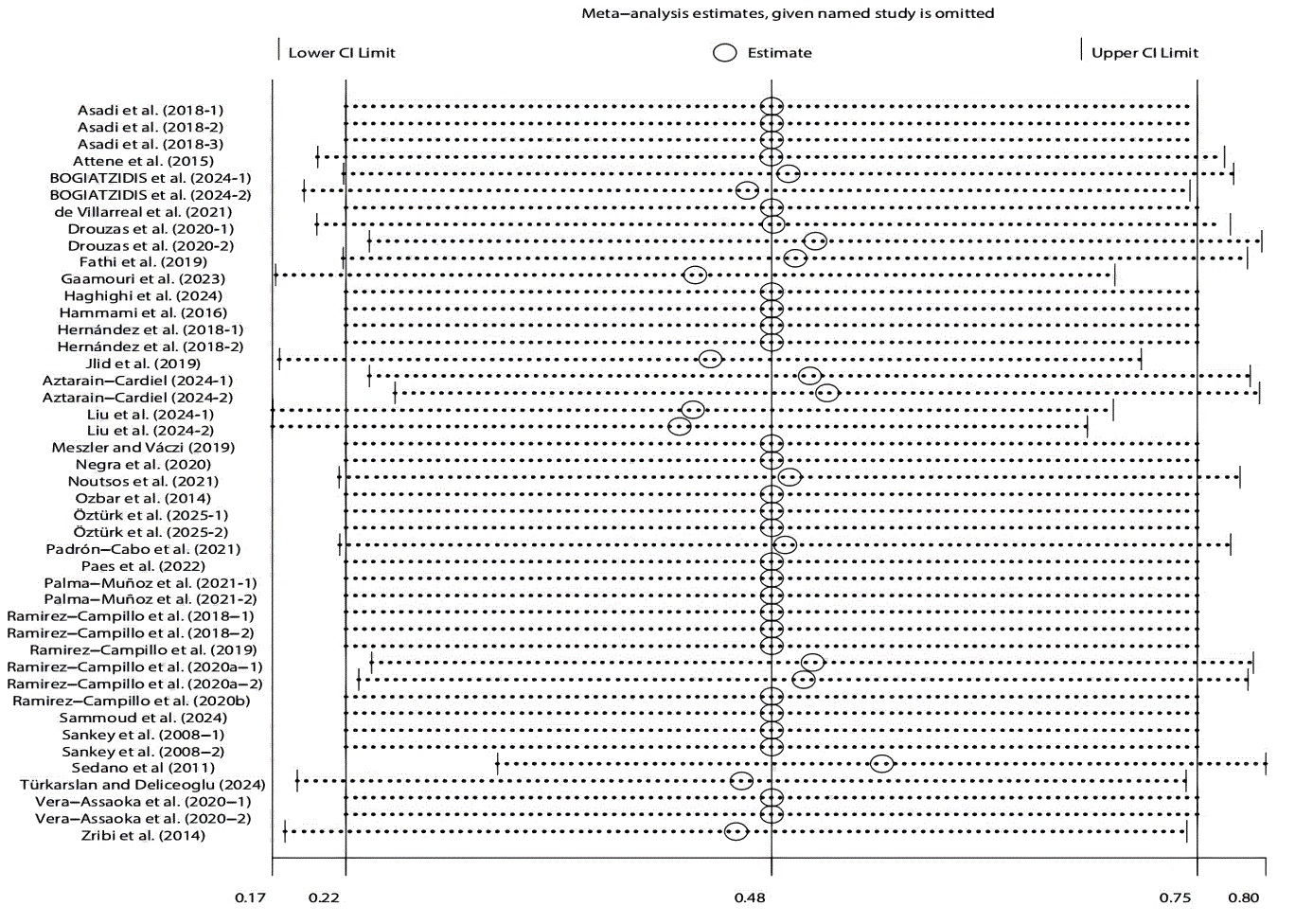

Supplement: Supplementary file 5 [file Image2.jpeg]

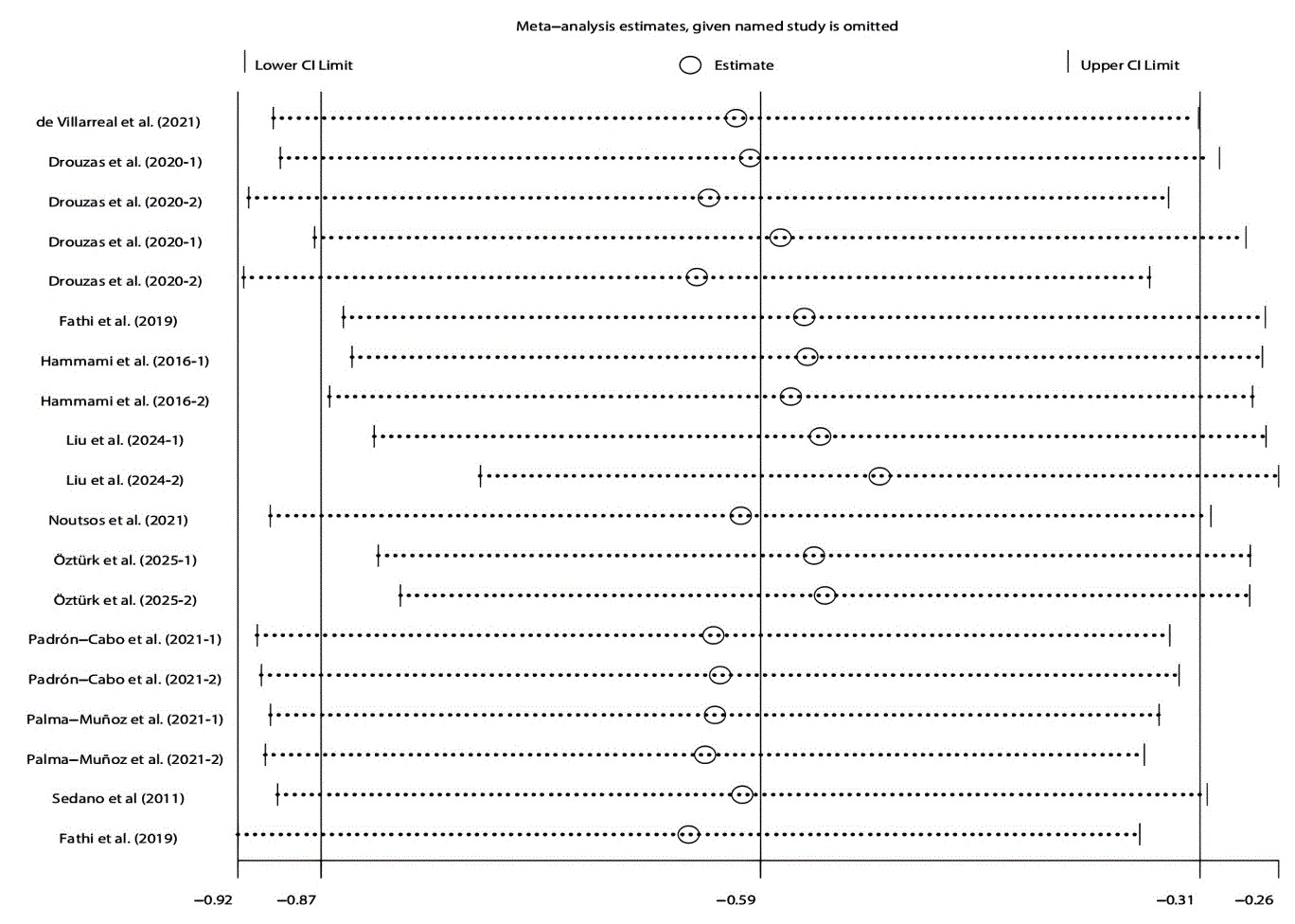

Supplement: Supplementary file 6 [file Image5.jpeg]

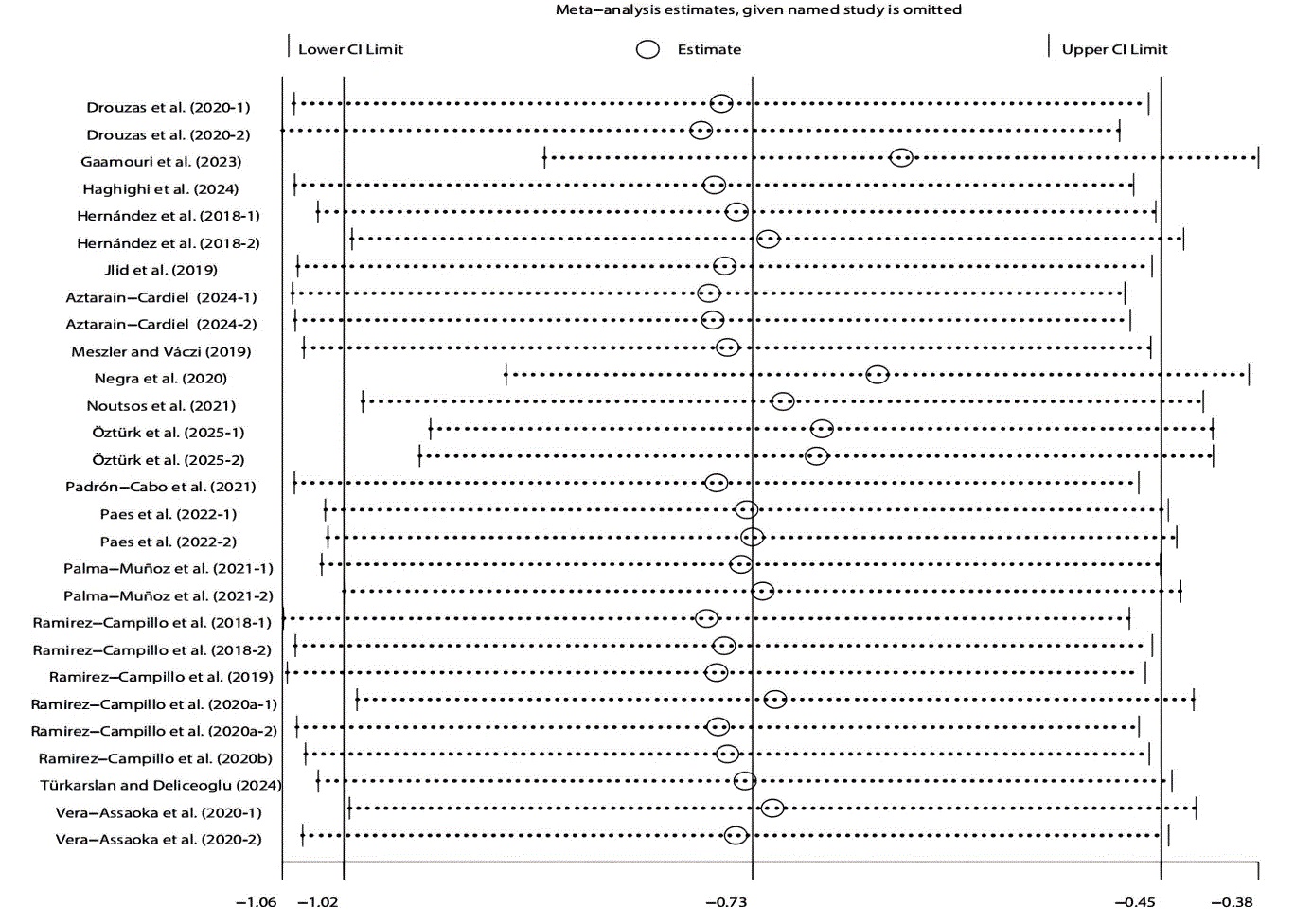

Supplement: Supplementary file 7 [file Image8.jpeg]

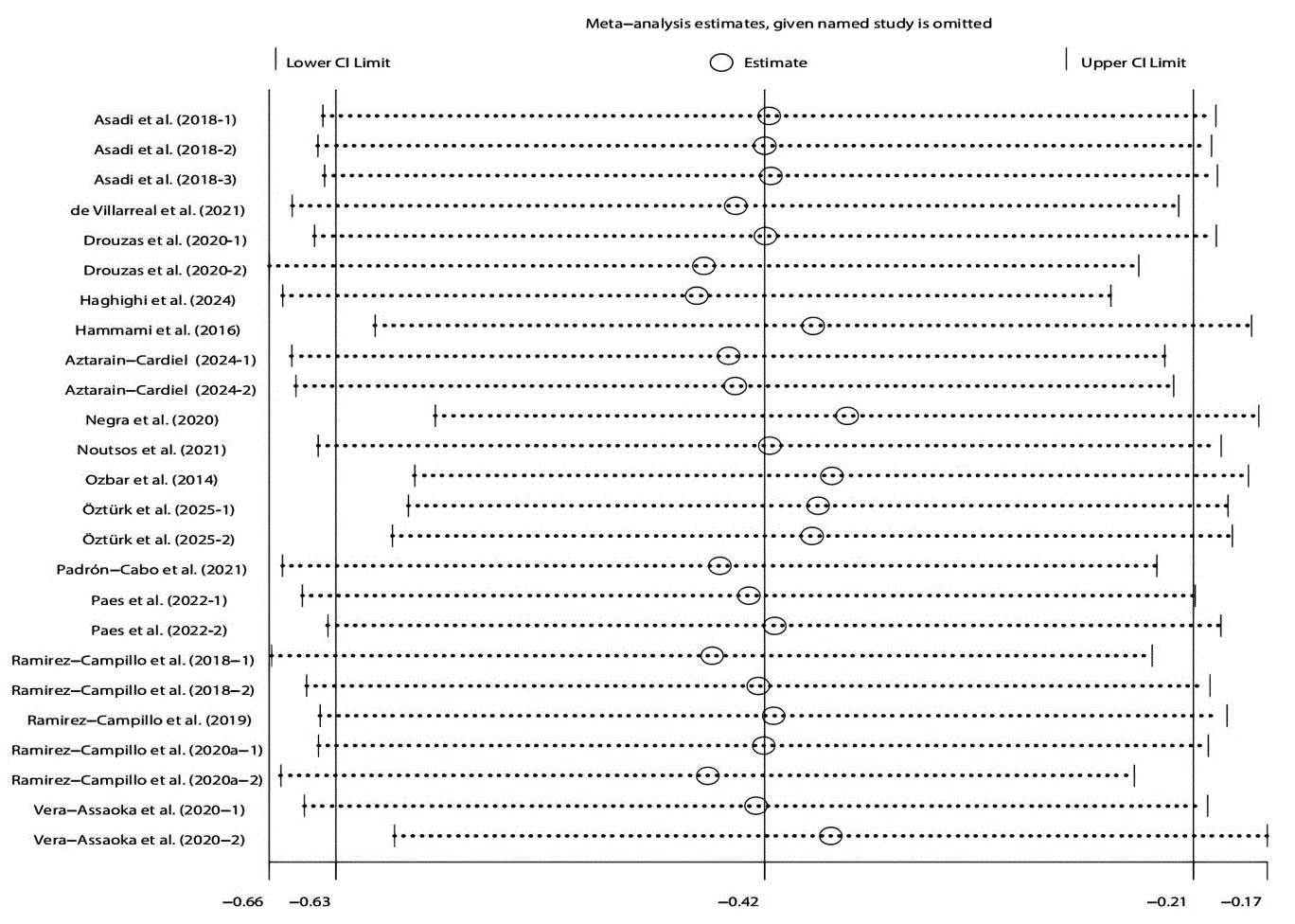

Supplement: Supplementary file 8 [file Image6.jpeg]
